# Epigenetic MLH1 silencing concurs with mismatch repair deficiency in sporadic, naturally occurring colorectal cancer in rhesus macaques

**DOI:** 10.1186/s12967-024-04869-6

**Published:** 2024-03-19

**Authors:** Simon Deycmar, Brendan J. Johnson, Karina Ray, George W. Schaaf, Declan Patrick Ryan, Cassandra Cullin, Brandy L. Dozier, Betsy Ferguson, Benjamin N. Bimber, John D. Olson, David L. Caudell, Christopher T. Whitlow, Kiran Kumar Solingapuram Sai, Emily C. Romero, Francois J. Villinger, Armando G. Burgos, Hannah C. Ainsworth, Lance D. Miller, Gregory A. Hawkins, Jeff W. Chou, Bruno Gomes, Michael Hettich, Maurizio Ceppi, Jehad Charo, J. Mark Cline

**Affiliations:** 1 Department of Pathology, Wake Forest University School of Medicine, Winston-Salem, NC USA; 2 Roche Postdoctoral Fellowship (RPF) Program, Basel, Switzerland; 3 Oregon National Primate Research Center, Oregon Health and Science University, Beaverton, OR USA; 4 School of Veterinary Medicine, University of California Davis, Davis, CA USA; 5 Department of Radiology, Wake Forest University School of Medicine, Winston-Salem, NC USA; 6 New Iberia Research Center, University of Louisiana-Lafayette, New Iberia, LA USA; 7 Caribbean Primate Research Center, University of Puerto Rico, Toa Baja, PR USA; 8 Department of Biostatistics and Data Sciences, Wake Forest University School of Medicine, Winston-Salem, NC USA; 9 Department of Cancer Biology, Wake Forest University School of Medicine, Winston-Salem, NC USA; 10 Center for Cancer Genomics and Precision Oncology, Wake Forest University School of Medicine, Winston-Salem, NC USA; 11 Department of Biochemistry, Wake Forest University School of Medicine, Winston-Salem, NC USA; 12 Roche Pharma Research and Early Development, Roche Innovation Center Basel, Basel, Switzerland; 13 Present Address: iTeos Therapeutics, Translational Medicine, Gosselies, Belgium; 14 Roche Pharma Research and Early Development, Roche Innovation Center Zurich, Zurich, Switzerland

**Keywords:** Colorectal cancer, Nonhuman primates, Mismatch repair deficiency, Epigenetic silencing, Translational oncology

## Abstract

**Background:**

Naturally occurring colorectal cancers (CRC) in rhesus macaques share many features with their human counterparts and are useful models for cancer immunotherapy; but mechanistic data are lacking regarding the comparative molecular pathogenesis of these cancers.

**Methods:**

We conducted state-of-the-art imaging including CT and PET, clinical assessments, and pathological review of 24 rhesus macaques with naturally occurring CRC. Additionally, we molecularly characterized these tumors utilizing immunohistochemistry (IHC), microsatellite instability assays, DNAseq, transcriptomics, and developed a DNA methylation-specific qPCR assay for MLH1, CACNA1G, CDKN2A, CRABP1, and NEUROG1, human markers for CpG island methylator phenotype (CIMP). We furthermore employed Monte-Carlo simulations to in-silico model alterations in DNA topology in transcription-factor binding site-rich promoter regions upon experimentally demonstrated DNA methylation.

**Results:**

Similar cancer histology, progression patterns, and co-morbidities could be observed in rhesus as reported for human CRC patients. IHC identified loss of MLH1 and PMS2 in all cases, with functional microsatellite instability. DNA sequencing revealed the close genetic relatedness to human CRCs, including a similar mutational signature, chromosomal instability, and functionally-relevant mutations affecting KRAS (G12D), TP53 (R175H, R273*), APC, AMER1, ALK, and ARID1A. Interestingly, MLH1 mutations were rarely identified on a somatic or germline level. Transcriptomics not only corroborated the similarities of rhesus and human CRCs, but also demonstrated the significant downregulation of MLH1 but not MSH2, MSH6, or PMS2 in rhesus CRCs. Methylation-specific qPCR suggested CIMP-positivity in 9/16 rhesus CRCs, but all 16/16 exhibited significant MLH1 promoter hypermethylation. DNA hypermethylation was modelled to affect DNA topology, particularly propeller twist and roll profiles. Modelling the DNA topology of a transcription factor binding motif (TFAP2A) in the MLH1 promoter that overlapped with a methylation-specific probe, we observed significant differences in DNA topology upon experimentally shown DNA methylation. This suggests a role of transcription factor binding interference in epigenetic silencing of MLH1 in rhesus CRCs.

**Conclusions:**

These data indicate that epigenetic silencing suppresses MLH1 transcription, induces the loss of MLH1 protein, abrogates mismatch repair, and drives genomic instability in naturally occurring CRC in rhesus macaques. We consider this spontaneous, uninduced CRC in immunocompetent, treatment-naïve rhesus macaques to be a uniquely informative model for human CRC.

**Graphical abstract:**

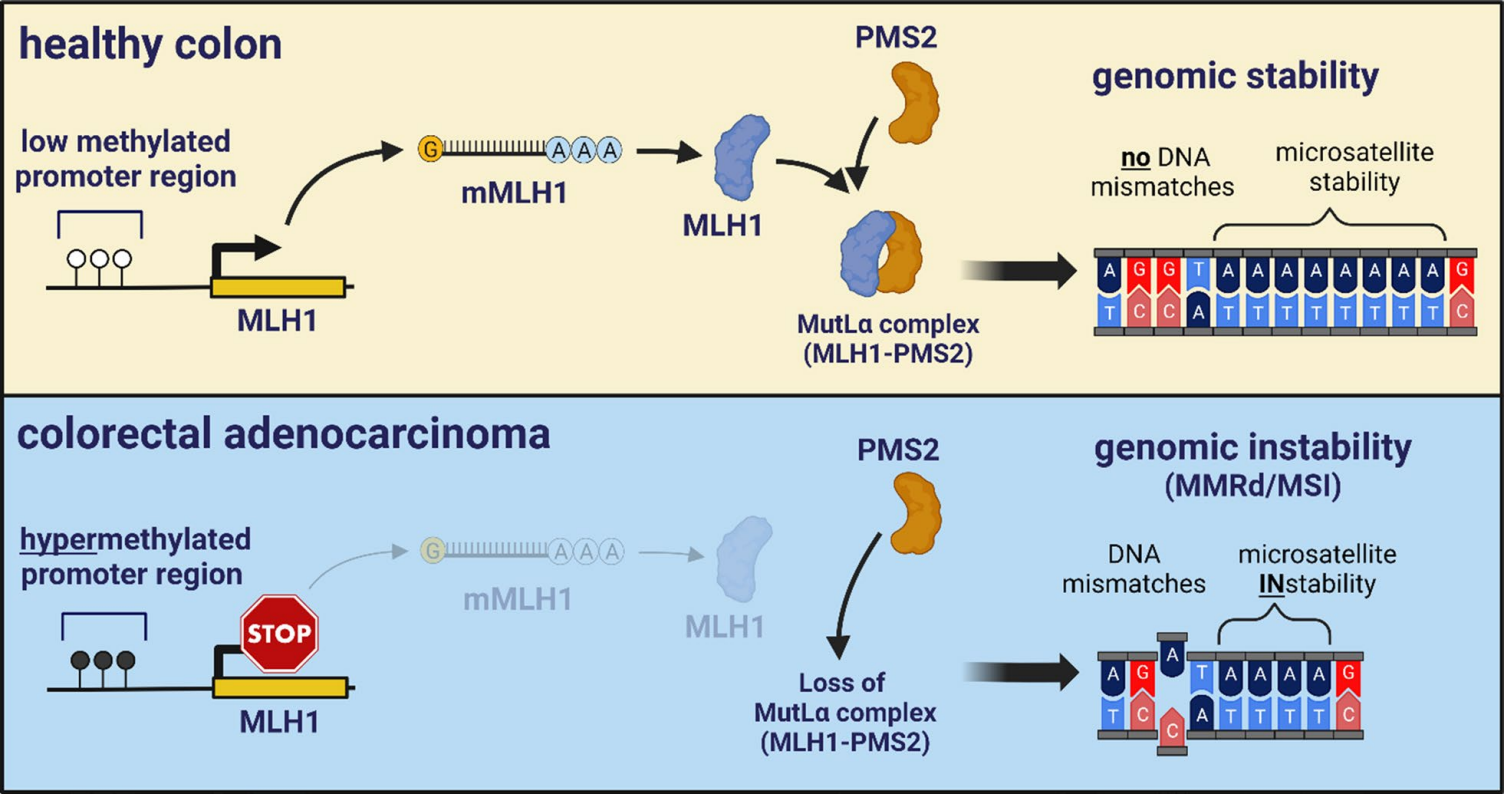

**Supplementary Information:**

The online version contains supplementary material available at 10.1186/s12967-024-04869-6.

## Background

Colorectal cancer (CRC) is the third most frequent cancer in humans with globally 1,880,000 estimated new cases and 916,000 estimated deaths in 2020 [1, 2]. While immunotherapy and immune checkpoint inhibitors (ICI) improved the outcome for certain cancer entities, the majority of CRCs are unresponsive [3]. This lack of progress is partially due to the scarcity of animal models that faithfully recapitulate human CRC and particularly reflects the complex interactions of the cancer cells, their hosting organism, and its immune system.

Rhesus macaques closely resemble human physiology but also have nearly identical immune cell subsets, regulatory mechanisms, and potential therapy targets as their human counterparts. These similarities include the natural and spontaneous development of CRC in rhesus macaques at similar life stages as observed in humans [4, 5]. Importantly, no induction/carcinogenic agents are administered to induce CRCs. The most common adenocarcinomas in rhesus macaques arise in the large intestine [4], and both sporadic and hereditary cases have been reported [6, 7].

The Primate Cancer Initiative at Wake Forest University School of Medicine (WFUSM) recruits cancer-bearing rhesus macaques for preclinical trials on novel cancer immunotherapies and combination therapies [5]. Multiple preclinical trials have been successfully performed in CRC- and breast cancer-bearing rhesus macaques [8–10]; generating valuable data on tumor targeting, pharmacodynamics, biomarkers, and informing proof-of-concept study design in humans.

Defective DNA mismatch repair (dMMR) results in microsatellite instability (MSI), is exhibited by 15–25% human sporadic CRCs, and is a primary driver of hereditary nonpolyposis colorectal cancer (HNPCC) [11]. dMMR and genomic instability result in a high tumor mutational burden (TMB), a positive predictor for ICI response [12] and an FDA-approved inclusion criteria for ICI administration [13]. Previous studies in pedigreed CRC-developing rhesus macaques [7] demonstrated a hereditary autosomal-dominant predisposition associated with MLH1 stop codons and deletions in the promoter region or MSH6 missense mutations, both key MMR proteins involved in human HNPCC/Lynch syndrome [11].

Epigenetic dysregulation such as DNA hypermethylation is a hallmark of cancer [14], but is not yet sufficiently investigated in rhesus CRCs. DNA hypermethylation occurs in CpG islands, conserved accumulations of CpGs abundant in the promoters of 60–70% of human genes [15], and generally reduces gene expression. Global epigenetic dysregulation results in the CpG island methylator phenotype (CIMP), which is reported in 15–20% of human CRCs, and causes the transcriptional silencing of tumor suppressor genes by promoter hypermethylation [16, 17]. In human CRC, extensive DNA hypermethylation, as illustrated by the CIMP phenotype, is positively associated with MLH1 promoter methylation, proximal/right-sided CRC location, microsatellite instability, BRAF mutations, and is generally limited to sporadic cases [18].

This study investigated colorectal carcinogenesis in a cohort of rhesus macaques as an animal model for human CRC by employing molecular assays to investigate MMR status, microsatellite instability, genetic mutations, transcriptional differences, and epigenetic alterations. Furthermore, we applied human tumor staging and grading guidelines to harmonize clinical information on our rhesus CRCs, including the use of clinical imaging procedures such as ^18^F-fluorodeoxyglucose positron emission tomography (^18^F-FDG-PET) scans and contrast-enhanced computed tomography (contrast CT).

### Methods

#### Experimental design

The spontaneous nature of rhesus colorectal cancer and the transfer of cancer candidates is an ongoing and less predictable process compared to other preclinical experiments. Thus, we performed clinical assessments on a rolling basis as cases were recruited, and molecular assessments were batched, and data presented here represent the information currently in hand.

### Animals

Our cohort consists of 24 Indian-origin rhesus macaques (18 females) that naturally developed CRC without the administration of carcinogens or induction agents. Candidates were identified and transferred to WFUSM from the Caribbean Primate Research Center [1], the New Iberia Research Center [1], and from the Oregon National Primate Research Center (ONPRC, 22) in the framework of the Primate Cancer Initiative. At all research facilities, commercially available monkey chow supplemented with fruit are fed to provide a complete and species-appropriate nutrient composition, which was continued at WFUSM. Animals from the ONPRC were pedigreed to confirm their outbred nature and out rule family clusters. Observed relationships were limited to 1 pair of half siblings (common sire) and 1 pair of half cousins (common granddam) within the ONPRC animals. Animals underwent a 60d quarantine and acclimatization period prior to any diagnostic procedures.

### Animal care and housing

Housing at WFUSM followed institutional and regulatory guidelines with species-appropriate daily environmental enrichment. Patients were observed twice a day for health, pain, and well-being and any irregularities were immediately reported to our veterinary staff. Monthly clinical assessments included hematology and blood chemistry, clinical physical examination, and abdominal ultrasound.

Standard diagnostic imaging consisted of ultrasound and plain CT and for selected cases contrast-enhanced CT and ^18^F-FDG-PET scans. Typical imaging findings that suggested invasive CRC include thickening of the submucosa and loss of normal colonic layering (ultrasound), colonic wall thickening (ultrasound, plain and contrast-enhanced CT), lumen constrictions (contrast-enhanced CT), or high focal metabolic activity (^18^F-FDG-PET).

To obtain wedge biopsies, animals were sedated with ketamine, intubated, and maintained on isoflurane anesthesia. Analgesia (e.g. buprenorphine) and antibiotics (e.g. ceftiofur) were administered by veterinarians according to guidelines and clinical observations. Postoperative monitoring was performed by veterinary staff and analgesia adapted based on the pain assessments and the animal’s well-being.

Upon elective or humane endpoint, a standardized necropsy protocol was performed, including gross and histologic examination of tumor tissue, adjacent lymphoid tissue, and all major organs.

All procedures were approved by the WFUSM Institutional Animal Care and Use Committee, in compliance with the U.S. Animal Welfare Act, The Guide for the Care and Use of Laboratory Animals, the Office of Laboratory Animal Welfare, and Public Health Service Policy. WFUSM is accredited by the Association for the Assessment and Accreditation of Laboratory Animal Care, International (AAALAC).

### Tissue processing and pathological cancer assessments

Tissues were fixed in neutral-buffered formaldehyde (10%), transferred to ethanol (70%), and paraffin embedded. H&E staining as well as immunohistochemistry were performed by the Comparative Pathology Laboratory Core Facility at WFUSM.

Histological assessments were performed by board-certified veterinary pathologists. We adapted staging and grading guidelines for NHP CRCs from established human CRC guidelines as published by the College of American Pathologists (CAP) [19] and the American Joint Cancer Commission AJCC (AJCC 8th Edition). While T staging of CRC was based on biopsy histology or necropsy; N- and M-staging were based on imaging, surgery reports, and complete organ assessment upon necropsy.

### Immunohistochemistry—MMR proteins

Immunohistochemistry (IHC) staining was performed using a Bond immunostainer (Leica BOND RX). The mismatch-repair IHC Panel included MLH1 (Agilent/Dako, IR079, monoclonal mouse anti-human MutL Protein Homolog 1, clone ES05), MSH2 (Agilent/Dako, IR085, monoclonal mouse anti-human MutS Protein Homolog 2, clone FE11), MSH6 (Agilent/Dako, IR086, monoclonal rabbit anti-human MutS Protein Homolog 6, clone EP49), and PMS2 (Agilent/Dako, IR087, monoclonal rabbit anti-human Posteiotic Segregation Increase 2, clone EPS1), as published in a previous study [6].

### Nucleic acid extraction

Tumor-free (healthy colon) and tumor-enriched DNA and RNA were derived from macrodissected FFPE sections (AllPrep DNA/RNA FFPE Kit, Qiagen) from all CRCs available at this study stage (CRC_1 to CRC_16). In case of living patients and limited tumor-free area within the biopsy, we extracted DNA from blood (Wizard Genomic DNA Purification Kit, Promega). All extracts were stored at – 80 °C.

### Microsatellite instability assay

We performed PCR of microsatellite loci: RheBAT25, RheBAT26, RheBAT40, RheD10S197, RheD18S58, and RheTGFβRII (Agilent Herculase II Fusion DNA polymerase, sequences see [20]). Amplicons were analyzed with an Agilent BioAnalyzer 2100 (DNA1000). Healthy and tumor electropherograms were overlayed and alternating amplicon lengths considered “instable”. CRCs were categorized “microsatellite-stable (MSS)”, “microsatellite-instable-low (MSI-low)”, and “microsatellite-instable-high (MSI-high)” when 0, 1, or ≥ 2 of 6 loci were determined as instable, respectively.

### Whole exome sequencing

Paired healthy and tumor-enriched DNA samples (see above in *Nucleic acid extraction*, CRC_1 to CRC_16) were submitted to the WFUSM Cancer Genomics Shared Resource. DNA was sheared (Covaris, USA), enriched by hybridization capture (xGen Exome Research Panel v2, Integrated DNA technologies, USA), and libraries prepared (KAPA HyperPrep, Roche, Switzerland) for sequencing on an Illumina platform (planned coverage: > 100 × healthy DNA, > 300 × tumor-enriched DNA).

100 bp PE reads were processed at the ONPRC using the mGAP analysis pipeline [21]. The Genome Analysis Toolkit Best Practices (GATK, RRID:SCR_001876, [22]) were implemented as follows: Reads were mapped to Mmul_10 [23] using BWA-MEM [24], duplicate reads marked with Picard 2.27 MarkDuplicates (RRID:SCR_006525, [25]), followed by GATK BaseRecalibrator. Somatic short variant discovery was performed with GATK v4.2.6 Mutect2 on tumor-normal pairs and filtered using the defaults for GATK FilterMutectCalls. SnpEff (RRID:SCR_005191, [26]) was used for impact prediction on protein coding genes.

Curation efforts focused on a gene panel which we derived from the COSMIC Cancer Browser (RRID:SCR_002270, [27]) by surveying the top mutations in versatile subtypes of human colorectal, breast, and hereditary cancers (complete 78 gene panel see Additional file 1: Table S1). Variants were validated with Integrative Genomics Viewer (IGV, Version 2.9.4, RRID:SCR_011793, [28]), COSMIC Cancer Browser [27], and mGAP [21].

Copy number alterations (CNA) analysis was performed following the GATK pipeline [29]. The targets of the IDT hybridization xGen Exome Research Panel v2 were lifted to Mmul_10 with UCSC liftover [30] to use as the target intervals for analysis. Intervals were preprocessed using the default padding of 250 bp. A CNV panel of normals was generated from the counts of the matched normal samples. Raw integers were counted with GATK CollectReadCounts, and standardized and denoised using the CNV panel of normals in GATK DenoiseReadCounts. GATK CollectAllelicCounts then tabulated counts at germline variant sites for the tumor and matched-normal samples. Germline variants were collected from the mGAP v2.3 vcf SNPS [21], subset to the preprocessed intervals. Next, GATK ModelSegments grouped together copy and allelic ratios that it determined are contiguous on the same segment. GATK defaults for ModelSegments gave a reasonable number of segments compared to changing defaults to increase or decrease number of segments. Finally, copy-neutral, amplified and deleted segments were called with GATK CallCopyRatioSegments and plotted with GATK PlotModeledSegments.

### RNA sequencing

cDNA libraries were prepared from total RNA (see above in *Nucleic acid extraction*, CRC_1 to CRC_16) using Illumina TruSeq Stranded Total RNA with Ribo-Zero Globin Preparation kit (Illumina). The library size distribution and quality were validated using an Agilent TapeStation, cDNA libraries quantitated with Qubit 3.0 (Thermo Fisher, USA), the libraries pooled, and sequenced on an Illumina NextSeq 500 (75 bp, SE) or Illumina NovaSeq 6000 (100 bp, SE).

Raw data processing was performed by the WFUSM Bioinformatics Shared Resource utilizing STAR sequence aligner (RRID:SCR_004463, [31]) and featureCounts [32]. Data analysis was performed utilizing R limma package [33, 34] and transcripts considered differentially expressed when a log(2)FC > │1│ and a p-value < 0.05 (adjusted for repeated testing) was observed. Plots with above mentioned cut-offs were generated with GraphPad Prism 9 (RRID:SCR_002798). Differentially expressed transcripts were compared to annotated human reference data utilizing Ingenuity Pathway Analysis (IPA, Qiagen, Version 73,620,684, RRID:SCR_008653).

### RT-qPCR

Macaque-specific TaqMan assays were used to determine mMLH1 (Rh0287580_m1) and mGAPDH (custom) as a reference. In compliance with MIQE guidelines, amplicon context sequences can be found in the supplements (see Additional file 1: Table S2). RT-qPCR was performed using a one-step RT-qPCR master mix (TaqMan Fast Virus 1-Step, ThermoFisher) and a qPCR instrument (QuantStudio 7, ThermoFisher) in a single well reaction following supplier’s guidelines. Ct values were processed in Excel and GraphPad Prism 9 was utilized for plots and statistical comparisons. The nonparametric Mann–Whitney U test was used for statistical comparisons and a p < 0.05 considered statistically significant.

### Methylation-specific qPCR & bisulfite conversion

Rhesus-specific primers and probes were designed for CACNA1G, CDKN2A, CRABP1, MLH1, and NEUROG1, CIMP markers used in humans [16]. We queried the region -1.500 bp to + 500 bp of the gene start (Mmul_10 [23]) utilizing IGV [28], compared sequence homology to a human reference (hg38) using BLAST [35], and sourced the obtained DNA sequence into MethPrimer 2.0 Primer Design web tool (The Li Lab at UCSF, RRID:SCR_010269, [36]). Taqman-based assays were designed and manually adapted to examine predicted CpG islands and assess 2–4 CpGs in a stretch of 20-30nt to achieve methylation-specific hybridization. In case of unavoidable CpG sites in the primer sequence (max. 1), we utilized both degenerate primers in an equimolar ratio to amplify this amplicon irrespective of the primer site’s methylation status. Input of bisulfite-converted DNA was normalized with a methylation-unspecific assay probing a CpG-free region in ACTB (Additional file 1: Table S3 for assay summary).

Extracted DNA (see above in *Nucleic acid extraction*, CRC_1 to CRC_16) was bisulfite-converted according to the manufacturer’s manual (EpiTect Bisulfite Kit, Qiagen), mixed with primers, probe, and master mix (EpiTect MethyLight PCR + ROX, Qiagen), and analyzed by qPCR (QuantStudio7 + QuantStudio Real-time PCR Software, ThermoFisher Scientific). Ct values were processed in Excel, and GraphPad Prism 9 utilized for plots and statistical comparisons (nonparametric Mann–Whitney U test; p < 0.05 was considered statistically significant).

In consideration of the tumor cell content in our macrodissected area (approximately 30–80%), we considered an increase of > 20% compared to paired healthy reference colon as a cut-off to assign a marker as “methylated” or “unmethylated” in CRC. Ultimately, CRCs were considered “CIMP negative”, “CIMP low”, and “CIMP high” when ≤ 2, 3, or ≥ 4 of the 5 investigated markers were methylated, respectively.

### In-silico prediction of transcription factor (TF) binding sites in promoter regions

We surveyed the promoter region (1500 bp 5’UTR) of MLH1, CACNA1G, CDKN2A, CRABP1, and NEUROG1 utilizing ConTra v3 (RRID:SCR_010814, [37]) for TF binding sites that (a) are conserved in humans and rhesus macaques and (b) overlapped with or are in close proximity to the probe-binding sequences used for methylation-specific qPCR. Predicted conserved TF binding were visualized with GraphPad Prism 9.

### Monte-Carlo simulations of intrinsic DNA topology upon DNA methylation

Intrinsic DNA topology (DNA shape) was examined by predicting spatial features such as minor groove width, helix twist, roll, and propeller twist utilizing Bioconductor package (RRID:SCR_006442) DNAshapeR (based on Monte-Carlo simulations of X-ray crystallography and NMR spectroscopy data [38]). We extracted the methylation-specific probe binding sequence ± 20 bp from IGV (Mmul_10, [28]) and predicted shape features for (1) all unmethylated CpGs and (2) methylated CpGs in the probe-binding region.

To link our experimental DNA methylation findings with in silico-generated findings, we focused on TF binding sites in the MLH1 promoter that overlapped with CpGs of experimentally confirmed methylation status. Binding site sequences were obtained from JASPAR [39], DNA shape features analyzed, and each nucleotide position across the TF binding site summarized as DNA shape percentiles. These reference shape profiles were overlayed with the shape features of the examined TF binding site, considering unmethylated or methylated CpGs.

### Comprehensive correlation analysis by Spearman correlation

We compiled numerous parameters of those CRCs with complete genomics datasets (CRC_1 to CRC_16) including age, sex, TNM staging, MSI status and No. of instable loci, TMB, KRAS mut, TP53 mut, APC mut, AMER1 mut, and the combined APC/AMER1 status, ARID1A mut, ALK mut, CIMP status, and No. of methylated marker, as well as numerical or structural CIN. We performed correlation analysis by nonparametric Spearman correlation (GraphPad Prism 10) and considered a p < 0.05 as statistically significant.

### Data and materials availability

DNA and RNAseq files were deposited in the Sequence Read Archive (SRA, RRID:SCR_001370) under BioProject PRJNA934967 and will be made available after a 1 year embargo from the day of publication. Further information on bioinformatics coding or applied cut-offs is available upon request. Any additional data is available from the corresponding author upon completion of a DTA.

## Results

### Rhesus CRC clinically and histologically resembles human CRC

**Fig. 1:**
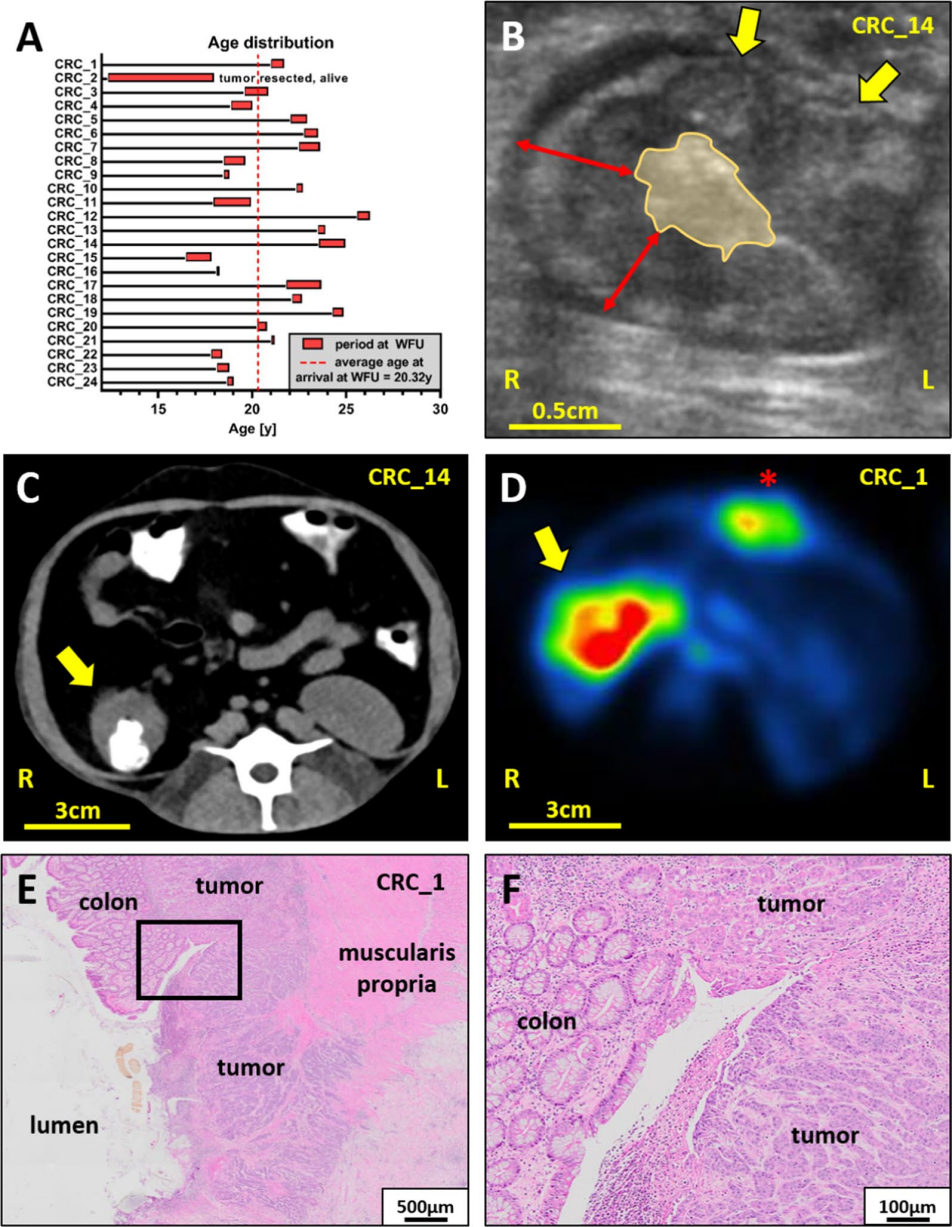
Colorectal cancer in rhesus macaques—age, diagnostic imaging and histopathology. **A** Age distribution of CRC-bearing rhesus macaques at the age of arrival at WFUSM and upon elective or clinical endpoints. **B** US imaging illustrates the loss of colonic wall architecture and replacement by nodular lesions (yellow arrows), the circumferential thickening of the colonic wall ranging from 0.5 to 1 cm (red arrows) and a constricted lumen (yellow area). **C** Iodine-based oral-contrast improves the accuracy of CTs by delineating the constricted lumen and colonic wall thickening (yellow arrow) at the ileocecal junction. **D** ^18^F-FDG-PET imaging highlights a circumferential region of high metabolic activity at the proximal colon (yellow arrow) and high metabolic activity at the healing laparotomy site (red asterisk). **E** + **F** H&E sections used for staging and grading illustrate the effaced colonic architecture and the penetration of both the circumferential and longitudinal muscle layers of the muscularis propria by the CRC. Note, the black rectangle in (**E**) highlights the area depicted with higher magnification in (**F**)

24 Indian-origin rhesus macaques with naturally occurring cancer (18 female, 6 male) were recruited and transferred to WFUSM on a rolling basis. This female predominance likely reflects the social structure of breeding colonies where females are retained for longer periods while young males are more frequently removed from breeding for research purposes. Their age at arrival ranged from 12.3 to 25.6y, averaging 20.32y (Fig. 1A). Imaging included ultrasound (US) (Fig. 1B), plain and contrast-enhanced CT (Fig. 1C), and ^18^F-FDG-PET (Fig. 1D). TNM staging followed guidelines of the American Joint Cancer Commission (AJCC 8th Edition) and the College of American Pathologists (CAP) [19], based on the extent of colonic wall penetration and local or distant metastasis (Fig. 1E and F and Table 1). A narrow majority of CRCs were confirmed by surgical biopsy (14/24) and all cases were right-sided in the proximity of the ileocecocolic junction or the hepatic flexure of the proximal, ascending colon.

With respect to imaging, US and CT scans were the primary screening method applied and available for the vast majority of animals (22/24, Table 1). Selected patients underwent ^18^F-FDG-PET scans (5/24) or contrast-enhanced CTs (3/24). Two CRC patients met humane endpoints during quarantine prior to diagnostic imaging. Notably, unexplained weight loss, unexplained hypoalbuminemia, or unexplained thrombocytosis in rhesus macaques 13 + years old were found to be warning signs indicating further screening with an abdominal ultrasound to exclude CRC in rhesus macaques. Thus, repeated US imaging provided the most promising and informative approach to recognize patients with at least stage 2 invasion (T2) and a loss of mural wall integrity due to infiltrating cancer cells. Fecal occult blood (FOB) can be used to corroborate imaging findings and was commonly observed (17/24) but was more common in later stages of disease, as was abdominal palpation of a mass. In addition to local progression, we also observed abdominal carcinomatosis (CRC_5), lymph node metastasis, or distant metastasis to spleen, lungs, uterus, or the spine. Interestingly, we rarely observed liver metastasis, which is frequently reported in humans. Moreover, the primary origin of rhesus CRCs in our cohort was within the vicinity of the ileocecocolic junction and the hepatic flexure in the right-sided ascending colon and therefore not as evenly spaced out in the ascending colon, transverse colon, descending colon, sigmoid colon, and rectum as described for human CRCs. We frequently observed clinically significant comorbidities such as anemia, weight loss and cachexia, hypoproteinemia, and hypoalbuminemia in CRC-bearing rhesus macaques, comorbidities also frequently described in human CRC patients. Overall, CRC mortality in rhesus macaques is commonly attributed to the growth pattern of the primary lesion, which frequently forms a dense fibrotic “napkin ring”-like lesion in the majority of cases (23/24) that obstructs the lumen and impairs GI function. In contrast, human CRCs more frequently appear polypoid on a stalk, and morbidities due to distant metastasis (such as liver) are the primary cause for patient decline.

**Table 1:** Clinical and pathological staging, grading, and observations in rhesus with naturally occurring CRC

ID	Sex	Age at		Diagnosis by	FOB	Available imaging	T	N	M	Grade	Fibrosis	Subtype	Localization / spread	Comorbidities
		Arrival	Death											
CRC_1	m	21.0	21.7	Biopsy	pos	US & CT & FDG-PET	3	1	0	2	pos	AdenoCa	Ileocecal junction, cecocolic junction, metastasis: tumor-assoc. LN	Weight loss, anemia, hypoproteinemia
CRC_2	f	12.3	n.A	Biopsy	pos	US & CT	3	0	0	2	pos	AdenoCa	Ileocecal junction, cecocolic junction	st.p.tumor resection, typhlocolitis
CRC_3	m	19.6	20.8	Necropsy	pos	US & CT	3	0	0	2	pos	AdenoCa	Cecocolic junction	Weight loss, azotemia
CRC_4	f	18.9	20.0	Biopsy	pos	US & CT	4	0	0	1	pos	AdenoCa	Ileocecal junction	weight loss, anemia, arthritis, thrombocytosis, lipoma
CRC_5	f	22.1	22.9	Biopsy	pos	US & CT	4	0	1	1	pos	AdenoCa	Ileocecal junction, spread to mesenteric tissue/jejunum, miliary omental carcinomatosis metastasis: spleen, uterus, ovary	weight loss, anemia, umbilical hernia
CRC_6	f	22.8	23.5	Biopsy	n.A	US & CT	3	1	1	1	pos	AdenoCa	Ileocecal junction (resected) tumor recurrence inflicting ovary, distant metastasis to adrenal gland, spine, extensive infiltration of vertebral body L2	st.p.tumor resection, hind limb paresis
CRC_7	m	22.5	23.6	Biopsy	pos	US & CT & FDG-PET	3	1	0	1	pos	Mucinous AdenoCa	Ileocecal junction metastasis: LN	Hypoalbuminemia
CRC_8	m	18.5	19.6	Biopsy	pos	US & CT & FDG-PET	3	1	0	1	pos	Mucinous AdenoCa	Ileocecal junction metastasis: LN	Anemia, hypoalbuminemia
CRC_9	f	18.5	18.8	Biopsy	pos	US & CT	3	1	1	1	pos	Mixed (mucinous/ AdenoCa/solid)	Ileocecal junction metastasis: LN, lung, omentum	Weight loss, anemia, hypoproteinemia
CRC_10	f	22.4	22.7	Biopsy	pos	US & CT	4	0	0	1	pos	AdenoCa	Ileocecal junction, local spread to mesentery	Acute gastric bloat, hypochromic-microcytic anemia, hypoalbuminemia, hypoproteinemia, thrombocytosis
CRC_11	f	17.9	19.9	Biopsy	n.A	US & CT	4	1	1	1	pos	AdenoCa	Ileocecal junction, metastasis: lung, liver, uterus, ax.LN, spleen, stomach, adrenal gland, pancreas, urinary bladder, ovary, omentum, bone (rib)	Weight loss, anemia, hypoalbuminemia
CRC_12	f	25.6	26.2	Necropsy	neg	US & CT & contrast-enhanced CT	3	1	0	1	pos	AdenoCa	Ileocecal junction, lymphatic invasion	Abdominal distensions, cecal obstruction, arthritis
CRC_13	m	23.5	23.8	Necropsy	n.A	US & CT	3	0	0	1	pos	AdenoCa	Ileocecal junction	Urinary retention due to chronic bladder atony, weight loss, arthritis, scoliosis, 0.25 × 0.1mm Meningioma
CRC_14	f	23.6	24.9	Biopsy	pos	US & CT & contrast-enhanced CT	2	0	0	1	neg	AdenoCa	Ileocecal junction	Hypoalbuminemia
CRC_15	f	16.5	17.8	Biopsy	pos	US & CT & contrast-enhanced CT	2	0	0	1	pos	AdenoCa	Ileocecal junction	Acute post-surgery complications, hypoalbuminemia
CRC_16	f	18.1	18.2	Necropsy	n.A	n.A	3	0	0	1	pos	AdenoCa	Ileocecal junction	Acute partial cecal obstruction, cachexia
CRC_17	f	21.8	23.6	Necropsy	pos	US & CT	3	0	0	2	pos	Mucinous AdenoCa	Cecocolic junction	Anemia
CRC_18	f	22.1	22.6	Necropsy	pos	US & CT	2	0	0	1	pos	AdenoCa	Ileocecal junction	Anemia
CRC_19	f	24.3	24.8	Biopsy	pos	US & CT & FDG-PET	3	0	0	1	pos	AdenoCa	Ileocecal junction, proximal colon	Anemia
CRC_20	f	20.3	20.8	Necropsy	pos	US & CT	2	0	0	2	pos	AdenoCa	Ileocecal junction	Anemia
CRC_21	f	21.0	21.2	Biopsy	n.A	n.A	3	1	0	3	pos	AdenoCa	Proximal colon, metastasis: mesenteric LN	Cachexia, anemia, esophageal hyperplasia due to chronic gastric reflux
CRC_22	m	17.8	18.4	Necropsy	pos	US & CT & FDG-PET	3	0	0	2	pos	AdenoCa	Proximal colon, small vessel invasion	Anemia
CRC_23	f	18.1	18.7	Necropsy	pos	US & CT	2	0	0	1	pos	AdenoCa	Ileocecal junction	Anemia
CRC_24	f	18.7	n.A	Biopsy	neg	US & CT	3	0	0	3	pos	Mucinous AdenoCa	Ileocecal junction	st. p. tumor resection
Average		20.3	21.3											

### MMR deficiency with MMR protein loss and MSI is a consistent feature in rhesus CRCs

**Fig. 2:**
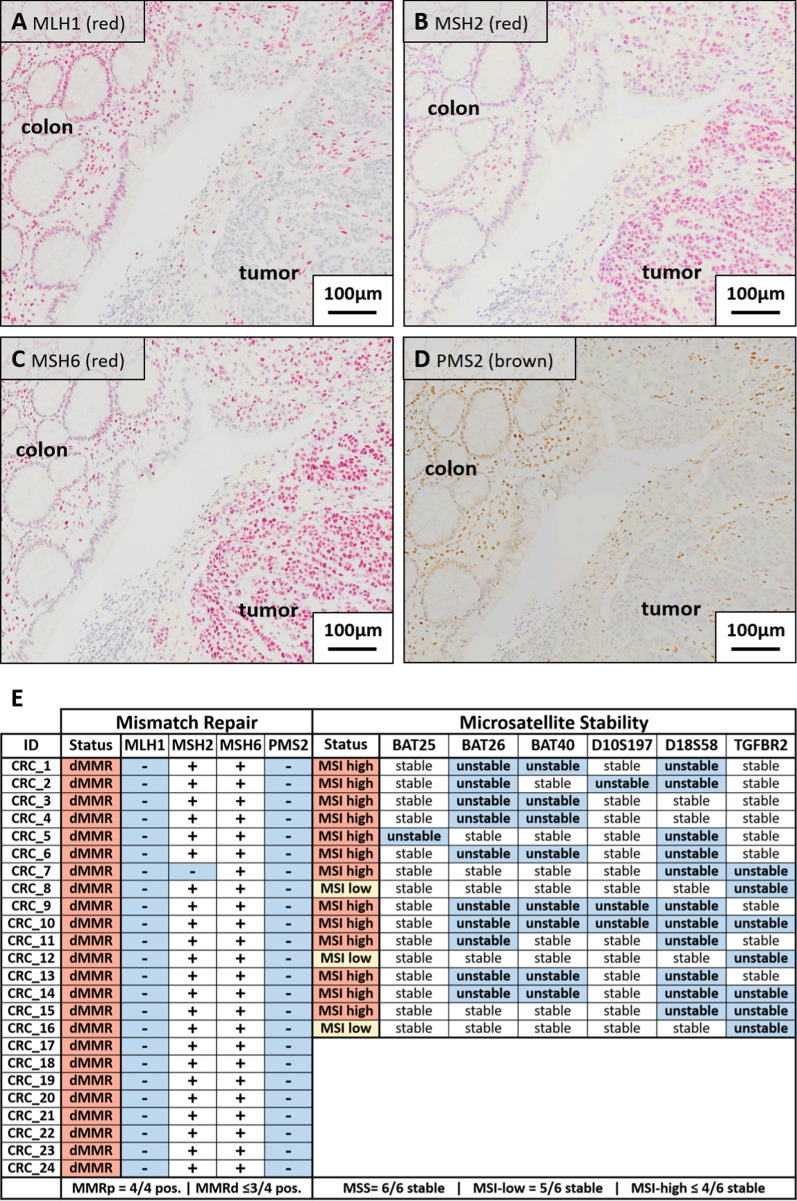
Mismatch repair deficiency due to concurring MLH1/PMS2 loss is a prevalent feature of naturally occurring CRC in rhesus macaques and results in functional microsatellite instability. Exemplary IHC staining of CRC_1 for MMR proteins **A** MLH1, **B** MSH2, **C** MSH6, and **D** PMS2. The consistent nuclear expression of these proteins in healthy colon and infiltrating lymphocytes served as an internal positive control. As clearly depicted, MLH1 and PMS2 staining was lost in the neoplastic cells but MSH2 and MSH6 were highly expressed. **E** This pattern of MLH1/PMS2 loss was observed cohort-wide in all 24 rhesus CRCs. MSH2 expression was maintained in 23/24 CRCs and MSH6 expression was present in all 24/24 CRCs. The emphasized mismatch repair deficiency translates to an instable genetic phenotype as corroborated by the widespread microsatellite instability. In summary, 13/16 CRCs were classified as MSI-high (≥ 2 unstable loci), 3/16 as MSI-low (1 unstable loci), and none as MSS

We performed IHC for key MMR proteins MLH1 (Fig. 2A), MSH2 (Fig. 2B), MSH6 (Fig. 2C), and PMS2 (Fig. 2D). Healthy cells adjacent to the tumors were consistently positive for all investigated MMR proteins (Fig. 2A–D) acting as an internal staining control as well as highlighting the consistent expression of MMR proteins. We observed cohort wide loss of MLH1 expression in all 24 rhesus CRCs (Fig. 2E) which was consistently accompanied by loss of PMS2 expression. This distinct pattern of MLH1/PMS2 double loss matches the IHC pattern associated with MLH1 abrogation in human CRCs and allows a distinctive delineation of cancer cell clusters and adjacent healthy cells [40, 41]. In contrast, all but one CRC (CRC_7) expressed MSH2 (23/24, 95.8%) and all strongly expressed MSH6 (24/24, 100%).

Functional genetic instability due to widespread mismatch repair deficiency was additionally corroborated by assessment of microsatellite stability in rhesus CRC samples present at this time (Fig. 2E). Thus, we performed MSI screening for 16 CRCs and identified 13 CRCs (81.3%) with ≥ 2/6 instable microsatellites and 3 CRCs (18.7%) with instability of 1/6 microsatellites being classified as MSI-high and MSI-low, respectively. No CRC was classified as MSS. The observed agreement between MMR status and microsatellite instability highlights the significance of alterations in this DNA repair pathway for sporadic colorectal carcinogenesis in rhesus macaques.

### Similar cancer-associated mutations, mutational signatures, and chromosomal instability are present in rhesus CRC as in human cancers, but MMR protein loss does not arise from genetic alterations

Somatic mutations called in rhesus CRC showed remarkable overlap with their human counterparts (Additional file 2: File S1), affecting the Wnt pathway (APC, AMER1), MAPKKK pathway (KRAS, NF1), AKT pathway (AKT1, PTEN), kinase pathway receptors (ALK, EGFR), epigenetic regulators (ARID1A, EP300), DNA repair (ATM, ATR), and cell adhesion (CDH1, PTPRT). We identified 304 non-synonymous and high-confidence mutations in our 78 gene hotspot panel of which 296 (97.4%) affected a similar amino acid as in humans. This observed protein homology exceeds the literature on DNA sequence homology of rhesus macaques and humans of 93.5% [42]; presumably reflecting the highly conserved nature of the examined cancer-associated gene panel.

Moreover, 69.4% (211/304) of mutations identified in rhesus macaques not only affected the same amino acids (AA) but also match the AA change reported in the human COSMIC data base. Notably, matching AA changes are more frequently observed for genes which are also more frequently examined in human cancers. As an example, there are 180,000, 280,000, and 200,000 COSMIC entries from human cancers for genes such as TP53, KRAS, or EGFR, respectively. In contrast, there are only 50,000 to 60,000 entries from less examined genes such as LRP1B, GRIN2A, and AMER1, potentially missing pathogenic variants we observed in our rhesus CRCs.

We contextualized the variant codon location using the amino acid sequence of the rhesus homologue of the human protein. Thus, we determined that 92.1% (280/304) of affected codons in rhesus are within ± 5 codons of the corresponding human codon coordinates (Additional file 1: Fig. S1), emphasizing an adequate spatial match of the codon coordinates in the rhesus and human reference genome annotations.

**Fig. 3:**
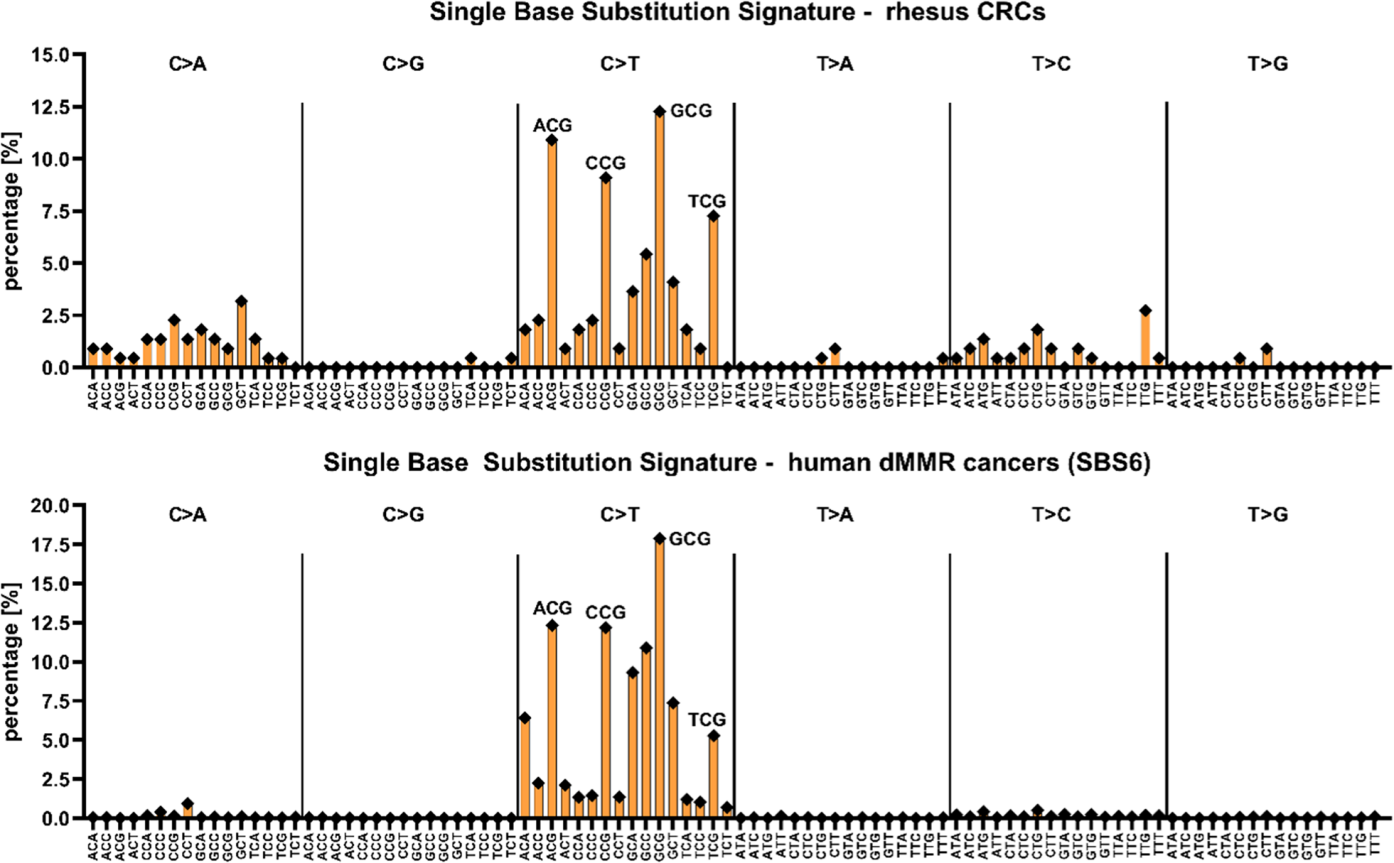
The mutational signature of rhesus CRCs is congruent to the human SBS6 signature with dMMR as proposed etiology. Most base substitutions observed in rhesus CRC are C > T transitions with a clear enrichment for ACG, CCG, GCG, and TCG. This pattern is highly similar to the human SBS6 signature [43], which is strongly associated with mismatch repair deficiency

Certain mutagenic mechanisms including defective DNA repair can result in distinct mutational signatures, resulting in 49 single-base substitution signatures (SBS) being determined in human cancers [43]. In rhesus CRCs, we observed a high frequency of C > T transitions, particularly within NCG trinucleotides which resembles the SBS6 signature, a signature associated with human dMMR tumors (Fig. 3). This corroborates our findings by IHC and MSI assays and supports the role of dMMR as major mutagenic driver in rhesus CRC.

Additionally to single base substitutions, we performed CNA analysis to probe for chromosomal instability (CIN), a common feature of human cancers (see Additional file 1: Fig. S2). We detected aneuploidies (7/16, numerical CIN), primarily affecting chr17 and chr19, as well as amplification or loss of large chromosomal compartments (12/16, structural CIN). Interestingly, chromosomal rearrangements could impact entire chromosomal arms, interchromosomal sections, or particularly accumulate at the chromosomal telomeric ends. Moreover, regions with varying allele frequencies can be identified in all examined rhesus CRCs, suggesting frequent loss-of-heterozygosity (LOH). These findings emphasize widespread CIN in naturally occurring rhesus CRCs.

Next, we assessed the affected genes and the functional impact of the called variants (see Table 2 and Additional file 1: File S1) and translated our information from DNA to protein level, e.g., we identified TP53 mutations such as p.R273*, the most frequently mutated TP53 codon in human cancers, and p.R175H, the most frequent TP53 mutation in human cancers. Moreover, 9/16 rhesus CRCs exhibited KRAS mutations including p.G12D (4/16 rhesus CRCs, most frequently affected KRAS codon and most frequent KRAS mutation in human cancers), p.G12S, p.G13D, p.A59T, p.Q61R, p.R68W, and p.A146T. Interestingly, only 2 BRAF missense mutations were observed (p.R356Q & p.R667Q) and 3 CRCs presented with a frameshift in BRAF in a G-repeat microsatellite (p.P399L_fs).

**Table 2:** DNA sequencing reveals similar tumor variants in rhesus and human cancers but cannot explain the observed cohort-wide loss of MMR protein expression

ID	TMB [mut/Mbp]		Somatic											Germline			
			APC	AMER1	KRAS	BRAF	TP53	ARID1A	ALK	MLH1	MSH2	MSH6	PMS2	MLH1	MSH2	MSH6	PMS2
CRC_1	40.71	TMB high		p.F173L_fs	p.Q61R			p.P1328R_fs p.H2020N									
CRC_2	42.66	TMB high		p.R630L	p.G12D			p.A282V p.G859E	p.R28S				p.E745K				
CRC_3	50.36	TMB high					p.M73W_fs	p.Q423K p.S748N	p.G902R								
CRC_4	19.71	TMB high	p.A2120T	p.Q978*	p.G12S p.G13D	p.R356Q				p.S404Y							
CRC_5	23.72	TMB high							p.G875R					p.R516W			
CRC_6	31.03	TMB high	p.R2759H			p.P399L_fs	p.R175H			p.R497G_fs			p.T680M				
CRC_7	13.38	TMB high	p.R1450*		p.A59T			p.A273P_fs									
CRC_8	15.52	TMB high	p.G721* p.T1556N_fs		p.G12D			p.G771S p.P1569L_fs									
CRC_9	41.38	TMB high		p.A36V p.K238N p.K260N_fs p.A622P_fs	p.G13D p.R68W	p.P399L_fs	p.M73W_fs p.A119V p.R175H p.R337H	p.F2142S_fs									
CRC_10	47.47	TMB high	p.L204I p.R2721H	p.F173L_fs	p.G12D p.A146T	p.P399L_fs	p.P142L	p.L2240F p.A2238S	p.Q500R								
CRC_11	19.74	TMB high			p.A146T												
CRC_12	32.17	TMB high	p.A1296T p.A1755V p.N1792M_fs	p.F173L_fs	p.G12D			p.R1278* p.D1851T_fs									
CRC_13	25.85	TMB high	p.N627L_fs	p.F173L_fs		p.R667Q	p.M73W_fs										
CRC_14	30.98	TMB high	p.Y796C p.R1687Q				p.R273*	p.P1569L_fs									
CRC_15	17.66	TMB high															
CRC_16	22.98	TMB high						p.P1408Q_fs									
	Ø 29.71		8/16	7/16	9/16	5/16	6/16	10/16	4/16	2/16	0/16	0/16	2/16	1/16	0/16	0/16	0/16

WES of rhesus CRCs and post-processing revealed 304 non-synonymous mutations in the most frequently affected genes of human cancers (most relevant shown here, for more details see Additional file 1: File S1). These variants affect tumor suppressor and oncogenes like APC, AMER1, TP53, KRAS, and ARID1A. 69.4% of these mutations are exactly as reported in human COSMIC data base. Interestingly, potentially pathogenic somatic or germline mutations of MMR proteins were seen in only a minority of cases

With respect to mismatch repair deficiency, we observed potentially pathogenic somatic variants in CRC_4 (MLH1 p.S404Y), CRC_6 (MLH1 p.R497G_fs & PMS2 p.T680M), and CRC_2 (PMS2 p.E745K). On a germline level, a single potentially pathogenic MLH1 variant was identified in one monkey CRC_5 (p.R516W). In conjunction with the absence of other germline mutations affecting MMR proteins, this supports a sporadic and not hereditary carcinogenesis. In conclusion, we cannot attribute the observed cohort wide loss of MLH1 and PMS2 protein to widespread pathogenic somatic mutations nor to a hereditary enrichment for germline variants.

On the other hand, WES confirmed the genetic instability as highlighted by a high tumor mutational burden. In detail, the average mutational load in rhesus CRCs was nearly 30 Mut/Mbp and can thus be considered TMB high (cut-off in humans: > 10Mut/Mbp [44]).

### Transcriptomics demonstrate similarly dysregulated pathways in human and rhesus cancers and confirm transcriptional suppression of MLH1 in rhesus CRCs

**Fig. 4:**
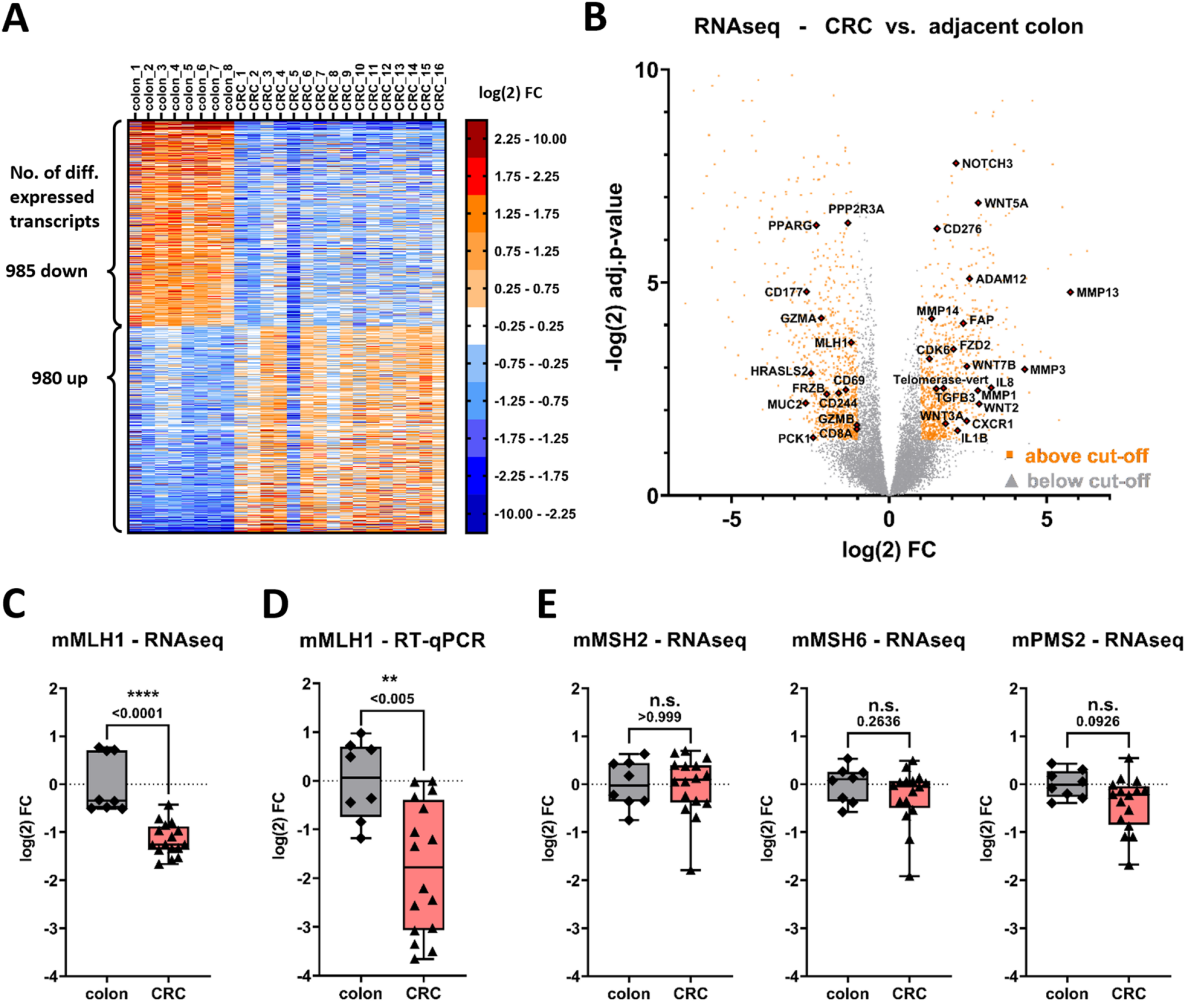
Transcriptomics reveals similar DEG signatures in rhesus and human CRCs and indicates transcriptional downregulation of MLH1. **A** RNAseq revealed 1965 significantly differentially expressed genes (985 up, 980 down) in rhesus CRC compared to adjacent healthy colon. **B** Genes frequently dysregulated in human cancers are similarly altered in rhesus CRC including upregulation of MMPs, FAP, telomerase, tumor-promoting or associated pathways and reduction of GZMA, FRZB, CD69, and PPARG. A statistically significant downregulation of MLH1 was determined in rhesus CRC by (**C**) RNAseq and corroborated by (**D**) RT-qPCR. **E** In contrast, no difference was observed for the other MMR proteins MSH2, MSH6, and even PMS2. Statistical analysis was performed by Mann–Whitney U test and a p < 0.05 was considered statistically significant

We performed RNAseq of CRC samples and unaffected colonic mucosa as a reference (coverage 25.3–55.5 M reads). We observed 1,965 differentially expressed genes (DEG) with a cut-off of log(2) FC >|1| and adjusted p-value < 0.05 (Fig. 4A). Proteins associated with tumor progression or a bad prognosis such as WNT5A, MMP13, MMP3, telomerase, and NOTCH3 were upregulated while proteins associated with immune cell infiltration or pathway inhibitors/tumor suppressors such as FRZB, CD177, PPARG, and HRASLS2 were downregulated (Fig. 4B). Importantly, our transcriptomics data close aligns with histopathologic observations and corroborates widespread tissue re-arrangement and fibrosis due to a highly activated cancer-associated stroma with substantial collagen deposition respectively an immunodepleted niche in rhesus CRCs (see Additional file 1: Fig. S3). This can be exemplified by the downregulation of CD8A, GZMA, and GZMB as well as NKG2D, CD209, and CD24, and a reduction in activation marker CD69 in rhesus CRCs. In contrast, we observed a significant upregulation of immune checkpoint B7-H3 (CD276) as well as TLR2 and its downstream targets IL8 and IL23A.

An unbiased comparison of our rhesus-derived CRC transcriptomics data to an annotated human reference database was performed using Ingenuity Pathway Analysis (IPA) and right-tailed Fisher’s Exact Test. In detail, IPA suggested solid tumor profiles such as “non-melanoma solid tumor” (p = 8.98*10^–63^) and “malignant solid tumor” (p = 1.01*10^–61^) within the “Cancer” and “Organismal injury & Abnormalities” category as top hits in respect of transcriptional overlap (Additional file 1: Fig. S4, A and B). This corroborates the transcriptional similarities in between rhesus and human cancers.

Most importantly, we observed a statistically significant downregulation of MLH1 by RNAseq (Fig. 4B, C), which could be corroborated by RT-qPCR (Fig. 4D). In contrast, other MMR proteins such as MSH2, MSH6, or PMS2 (Fig. 4E) were not differentially expressed comparing rhesus CRC and adjacent colon. These findings (a) emphasize MLH1 and not PMS2 as the key driver of dMMR and (b) points towards an epigenetic or transcriptional mechanism to induce dMMR at the intersection of genetics and protein levels.

### Hypermethylation of the MLH1 promoter region is observed in all investigated rhesus CRCs irrespective of CIMP status

**Fig. 5:**
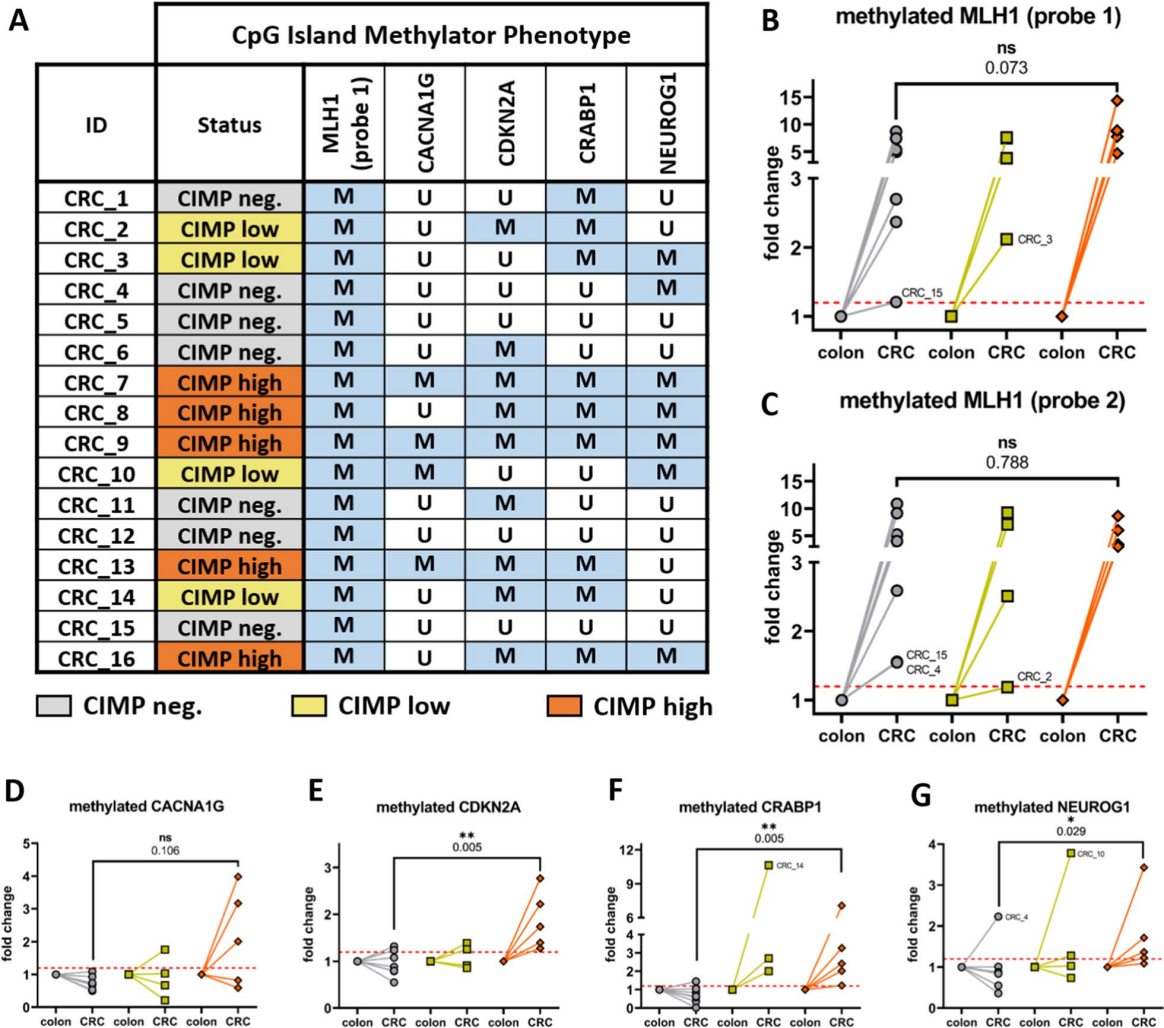
MLH1 hypermethylation occurs in all examined rhesus CRCs and is independent of the more global CIMP status. A marker was considered hypermethylated in CRC upon an increase of 20% in DNA methylation (red dotted line) in comparison to paired healthy colon as reference. **A** As a result, 5/16 CRCs were considered CIMP-high (orange, ≥ 4/5 methylated marker), 4/16 CRCs CIMP-low (yellow, 3/5 M), and 7/16 CRCs CIMP-negative (gray, ≤ 2/5 M). Interestingly, the MLH1 promoter region was hypermethylated in the entire CRC cohort (**B**, MLH1 probe 1) which was corroborated by an independently designed second probe (**C**, MLH1 probe 2). Importantly, this striking hypermethylation of MLH1 was observed in all CRCs and independent of the more global CIMP status. In contrast to MLH1, the other CIMP markers **D** CACNA1G, **E** CDKN2A, **F** CRABP1, and **G** NEUROG1 followed a clear trend and delineated CIMP high CRCs with higher methylation levels from CIMP negative tumors. Nonparametric Mann–Whitney U test was used for statistical comparisons and a p < 0.05 considered statistically significant

During design of rhesus-specific and DNA-methylation-specific qPCR assays, we utilized sequence alignment by BLAST and confirmed a consistent sequence homology of the rhesus and human promoter regions of MLH1, CACNA1G, CDKN2A, CRABP1, and NEUROG1 of 95.31%, 96.30%, 95.36%, 92,64%, and 95.00%, respectively. Interestingly, DNA methylation within the MLH1 promoter region was statistically significantly elevated in rhesus CRCs compared to adjacent healthy colon (Fig. 5A and B, Additional file 1: Fig. S5A). Importantly, this observation was corroborated by a second probe (Fig. 5C and Additional file 1: Fig. S5B), examining a different set of CpG’s in the MLH1 promoter which confirmed ubiquitous MLH1 promoter hypermethylation in all examined CRCs (16/16).

In contrast, DNA methylation of CACNA1G, CDKN2A, CRABP1, and NEUROG1 was not statistically significantly changed in a cohort wide assessment (Additional file 1: Fig. S5C–F). Hence, in accordance with human literature, we investigated the individual normalized paired tumor/colon samples and grouped them according to CIMP status (Fig. 5A). We observed a CIMP-high status (≥ 4/5 markers methylated) in 5/16 investigated rhesus CRCs (31.3%), a CIMP-low methylation level in 4/16 CRCs (25%), and a CIMP-negative status in 7/16 CRCs (43.8%). After grouping by the CIMP status, we observed no statistical difference in CIMP high versus CIMP negative CRCs for CACNA1G (Fig. 5D,  p = 0.106) but determined statistically significantly higher methylation levels for CDKN2A (Fig. 5E,  p = 0.005), CRABP1 (Fig. 5F. p = 0.005), and NEUROG1 (Fig. 5F, p = 0.029) in CIMP-high CRCs.

In summary, MLH1 hypermethylation was observed in all investigated rhesus CRCs and could be corroborated by a second assay but despite an overrepresentation of CIMP-positive rhesus CRCs compared to human CRCs, there is no general hypermethylated phenotype involved in epigenetic silencing of tumor suppressor MLH1.

To bridge the observed promoter hypermethylation and gene expression, we predicted conserved TF binding sites in the examined promoters of humans and rhesus macaques utilizing ConTra v3 [37], and modelled the impact of DNA methylation on DNA shape as mechanism of gene modulation.

We identified a high density of conserved TF binding sites in the examined CpG islands including binding motifs of SP1, SP2, TFAP2A (AP2α, MA0003.4), KLF5, E2F, and E2F1 (Additional file 1: Fig. S6), supporting the high relevance of these gene promoter regions for expressional modulation.

In a second step we performed Monte-Carlo simulations to model the impact of DNA methylation on DNA topology (minor groove width, helix twist, propeller twist, roll) in the probed regions as a potential mechanism for altered protein/DNA interactions. Simulations confirmed the substantial alterations of DNA topological profiles upon CpG methylation, particularly lowering the propeller twist and increasing the roll profiles in contrast to only marginally affecting minor groove width and helix twist (Additional file 1: Fig. S7), matching available literature [45].

Importantly, our qPCR-probed sequence in MLH1 (probe 1) overlaps with a predicted TFAP2A (AP2α) binding site, allowing us to dissect the impact of experimentally confirmed DNA hypermethylation on the DNA shape in this particular binding motif (Additional file 1: Fig. S8). The unmethylated sequence, as predominant in healthy rhesus colon, exhibits a similar DNA shape profile in comparison to the propeller twist and roll profiles of known TFAP2A binding sequences (n = 15,967, JASPAR). In contrast, CpG methylation in the binding motif, as experimentally demonstrated in rhesus CRC, causes a substantial departure from the topological profile of known TFAP2A binding sites. Thus, similar changes can be expected in other TF binding sites throughout TF binding motif-rich promoter regions such as MLH1 and provide a potential mechanism for TF binding interference by DNA hypermethylation.

Lastly, we performed correlation analysis (see Additional file 1: Fig. S9) using nonparametric Spearman correlation and identified multiple statistically significantly correlated parameters. In brief, we found a positive correlation of age at arrival with APC mutations, of TP53 mutations with the No. of instable MSI loci and TMB, TMB with the No. of instable MSI loci and ALK mutations, and interestingly ARID1A mutations with CIMP positive status. Importantly, the connection of TP53 with higher TMB due to deregulated genome maintenance is similarly described in human cancers [46], as is the correlation of ARID1A mutations and CIMP positivity [47].

## Discussion

We deployed comprehensive state-of-the-art clinical methods and molecular assays to perform clinical staging and grading and a deep molecular characterization including DNAseq, transcriptomics, and epigenetic assessments. This elucidated the macroscopic, microscopic, and molecular pathology of carcinogenesis in naturally occurring CRC in rhesus macaques.

These efforts highlight the close similarities, including comorbidities such as anemia secondary to gastrointestinal blood loss, but also differences such as the excess of right-sided CRCs occurring at the ileocecocolic junction and the proximal ascending colon. We further illustrated that local invasion of rhesus CRCs, frequently presenting as constricting napkin-ring-like lesions, can cause mortality via the primary lesion while human mortality is more frequently attributed to distant metastasis affecting liver or lung [48]. Moreover, the high abundance of lesions in the right-sided, proximal colon in rhesus macaques suggests a more singular disease origin than observed in human CRC patients where lesions in the proximal colon, distal colon, and rectum are more evenly distributed [49]. A potential explanation might be the result of a more uniform plant-based animal feed for rhesus macaques in contrast to humans with exposure to red or processed meats, alcohol, and cigarette smoke as established risk factors of CRC [1]. Alternatively, differences in behavior and lifestyle, microbiome, or a genetic respectively evolutionary component might play a role in the observed high abundance of proximal CRC in rhesus macaques. Importantly, pathological features such as histopathologic appearance, local fibrosis, mucin production, and malignant infiltration of the colonic layers are similar to human CRCs.

Another finding was the ubiquitous loss of MLH1 protein in conjunction with PMS2 absence, implying dMMR and suggesting a potential use as surrogate biomarker for rhesus CRC diagnostics due to its clear-cut separation of cancer cell clusters and healthy tissue. In human CRC, double loss of MLH1/PMS2 can be frequently attributed to MLH1 abrogation, while MLH1 protein is often preserved in cases of PMS2 abrogation [40, 41]. Hence, MLH1 loss can be similarly expected as the major driver of dMMR in rhesus CRC. Interestingly, the complete penetrance of MLH1 loss respectively dMMR in our rhesus CRC cohort drastically exceeds reports from human sporadic CRC of 15–25% dMMR/MSI [11, 48]. Importantly, dMMR could be confirmed on a functional level by the consistently observed microsatellite instability, the high TMB, and a similar mutational signature in rhesus CRCs.

This is the first study performing whole-exome DNAseq on rhesus CRCs, providing an unparalleled view on the genetic landscape of rhesus cancers. DNAseq confirmed genetic instability as illustrated by a mean TMB of nearly 30 Mut/Mbp; well above the threshold for human solid cancers to be considered TMB high (> 10 Mut/Mbp) [44]. Even more so, the mutational signature of rhesus CRCs closely matches a human cancer signature with mismatch repair deficiency as a proposed etiology, supporting dMMR as the major driver of mutagenesis and carcinogenesis. Furthermore, we confirmed both numerical and structural CIN of rhesus CRCs as emphasized by aneuploidies particularly of chr 17 and chr19 and amplification respectively loss of entire chromosomal regions as well as frequent LOH. In humans, dMMR, MSI, and a high TMB are positive predictors of response to ICI by drugs such as pembrolizumab or atezolizumab [50, 51]. Therefore, a similarly beneficial response profile to ICI as described in human can be anticipated in dMMR/MSI/TMB_high_ rhesus CRCs. As a result, multiple trials assessing immune checkpoint blockade alone or in combination with radiotherapy are currently being performed in rhesus macaques with naturally occurring dMMR CRCs. The primary aim of these biomarker-focused trials is the assessment of the peripheral response in circulating PBMCs and axillary lymph nodes respectively regionally in the tumor and draining mesenteric lymph nodes [52, 53].

With respect to the genetic background of rhesus CRCs we were able to highlight the close relatedness to human CRCs as emphasized by KRAS, TP53, APC, AMER1, ARID1A, and ALK mutations. Nearly 70% of the mutations observed in rhesus CRCs are as recorded in the human COSMIC cancer database. Furthermore, we were able to demonstrate that somatic and germline mutations in MMR proteins such as MLH1 and PMS2 can only be detected in a small minority of dMMR rhesus CRC cases. Consequently, we were able to exclude genetic mutations as source of the observed cohort wide dMMR and microsatellite instability.

Following DNAseq we performed RNAseq to elucidate the transcriptional landscape of rhesus CRC. Our work again highlighted the close relatedness of rhesus and human CRCs as emphasized by upregulation of MMPs, telomerase, WNT pathway key proteins, or FAP and downregulation of cancer suppressive mechanisms such as cytotoxic T cells and other immune cell populations and Wnt inhibitory proteins. Importantly, transcriptomic data suggests a generally immunosuppressed niche and a substantial increase in CAF markers, collagen and MMP production in rhesus CRCs, closely matching the histopathological findings upon microscopic evaluation. Furthermore, the DEG profile in rhesus CRCs closely overlapped with human annotated transcriptional data, resulting in similar and correct appraisement of neoplasm and solid/GI malignancy as most probable disease.

Of highest significance is the finding of a cohort-wide transcriptional suppression of MLH1 while no statistically significant changes were observed for MMR proteins MSH2, MSH6, or PMS2. This MLH1-exclusive transcript suppression was furthermore corroborated by RT-qPCR as a validation read-out. As a result, we consider loss of MLH1 transcription as causative for the absence of the MutLA complex (MLH1/PMS2) and the consequently observed dMMR, microsatellite instability, high tumor mutational burden, and mutational profile.

To further investigate the source of transcriptional suppression of MLH1, we assessed the DNA methylation in CpG islands of the promoter region of MLH1 and genes utilized as marker for CIMP status in human CRCs. Indeed, comparing rhesus CRC and healthy adjacent colonic tissue, we observed statistically significantly higher levels of DNA methylation in the MLH1 promoter region of CRC samples. These findings were verified by a second, independently designed qPCR probe assessing different CpG’s in the MLH1 promoter region. Interestingly, MLH1 hypermethylation occurred irrespective of the more global DNA methylation levels as described by the CIMP status. Specifically, we determined a CIMP positive background in about half the rhesus CRCs, but all of them exhibited DNA hypermethylation in the MLH1 promoter region, suppressing MLH1 transcription and ultimately MLH1 protein levels.

We furthermore utilized in silico tools to predict conserved TF binding sites in CpG islands in humans and rhesus macaques. The high number of conserved TF binding motifs supports the importance of these regions for modulation. Moreover, Monte-Carlo simulations allowed us to predict the impact of DNA methylation on DNA shape profiles in the promoter regions. As a result, we highlighted the impact of DNA methylation on DNA topology in all six regions, primarily affecting propeller twist and roll and only marginally altering minor groove width and helix twist. Additionally, we were able to link our experimental results from DNA-methylation-specific qPCR probes and in silico simulations at a TFAP2A (AP2alpha, MA0003.4) binding site that coincided with our MLH1 (probe 1) binding sequence. As a result, we demonstrated that the topological profile of the unmethylated region closely overlaps with those of known TFAP2A binding sites (n = 15,967). In contrast, methylation within the binding motif, as detected ubiquitously in rhesus CRCs, drastically alters the DNA shape, inducing a substantial departure from the topology of known TFAP2A binding sites. These findings illustrate the high impact of DNA methylation on regulatory elements, such as TF binding sites within CpG-rich promoter regions, and ultimately gene modulation.

Notably, there is a broad regulatory network responsible for directing DNA methylation in gene promoter regions. As an example, DNA methyl transferases such as DNMT1, DNMT3a, and DNMT3b are known to interact with a variety of transcription factors including the above mentioned TFAP2A as well as E2F5, ID2, MSX1, SP1, TP53, or NFκB-p50 [54, 55]. Alternatively, long noncoding RNAs are also shown to modulate DNA methylation by impacting the global methylome or directing recruitment of DNA methyltransferase to target genes [56–59]. Hence, versatile tissue, spatial, or lifestyle factors might be involved in triggering signaling cascades that ultimately lead to DNA hypermethylation and epigenetic silencing of particular gene promoters.

On a clinical level, 61% of CIMP positive tumors in humans occur in females, in the right-sided, proximal colon (59%), present with higher frequencies of MSI (36% high, 12% low, and only 52% MSS) and MLH1 methylation (39%) as well as frequent BRAF mutations (73%) but seldomly KRAS mutations (only in 10%) [18]. This pattern of concurrence of BRAF mutations, CIMP high, and MLH1 methylation in sporadically MSI CRCs of right-sided primary location is reported consistently in human literature [60, 61]. Additionally, MSI and KRAS mutations are frequently described as mutually exclusive [62].

In agreement with these statements, somatic MLH1 hypermethylation is reported in 91.9% of MSI/BRAF_mut_ but interestingly also in 61.7% of MSI/BRAF_wt_ human CRCs [63], the latter being the major phenotype in our rhesus CRCs. Unfortunately, this prior report did not address the KRAS or CIMP status. Another study investigating MLH1 hypermethylated/BRAF_wt_ CRCs reported KRAS mutations in 31% and right-sided origin in 86% of cases [64]. Our findings in rhesus CRCs highlight a similar tendency for MLH1 hypermethylated/primarily BRAF_wt_ rhesus CRCs with KRAS mutations observed in 56% and right-sided origin in 100% of the cohort.

As a perspective, our model of dMMR CRC originating from epigenetic silencing of MLH1 by promoter hypermethylation provides a unique opportunity to investigate the impact of FDA-approved methyl transferase inhibitors such as azacytidine or decitabine. These nucleoside analogues are described to restore MLH1 expression by promoter demethylation [65] and therefore probably re-establish mismatch repair proficiency. On the other hand, current approaches actually favor the induction of dMMR to increase TMB and neoantigen burden of tumors as being positively associated with response to immunotherapy [66–69]. Moreover, promising new small molecules targeting WRN and exhibiting synthetic lethality in dMMR/MSI cancers could be expected to work similarly efficient in our dMMR rhesus CRCs [70].

Ultimately, the distinct phenotype of sporadic MLH1 hypermethylation and dMMR CRCs in rhesus macaques provides an unprecedented opportunity to investigate the co-evolution of cancer in primates. While the overlaps with human CRCs are striking and range from similar mutational signatures, cancer-associated mutations, transcriptional profiles, clinical appearance, and even correlation of particular parameters such as ARID1A mutations with CIMP positivity, we also observe a disparity with respect to an overrepresentation of KRAS mutations and an underrepresentation of BRAF mutations (particularly V600E) in respect to human CRCs of this phenotype. As an outlook, a deepened knowledge on the evolutionary conserved or different alterations within rhesus and human cancers could highlight shared or human-exclusive cancer weak points that are potentially targetable by approaches such as novel kinase inhibitors, anti-sense oligonucleotides, or rational modulation of the tumor microenvironment or microbiome to name a few. We therefore believe that our study provides a strong rational and proof-of-concept for extensive comparative analysis of naturally occurring cancers in rhesus macaques including genomics and transcriptomics. Thus, an expansion of our research in the future might facilitate the investigation of our “last common cancer” and open doors towards a deeper understanding of not only cancer evolution in an individual but the evolution of cancer in the entire order Primates.

## Conclusions

CRCs in rhesus macaques can be similarly characterized on a clinical, microscopic, and molecular level as performed in human patients and closely resemble human CRCs with respect to affected pathways, observed DNA mutations, and clinical progression. The naturally occurring carcinogenesis accompanied by the treatment-naïve nature of these patients, and an unaltered immune system makes these CRC-bearing rhesus macaques an outstanding model for evaluating human cancer immunotherapy.

### Supplementary Information

**Additional file 1: Table S1.** 78 gene hotspot panel applied for tumor variant calling in rhesus CRCs. **Table S2.** RT-qPCR – probe and amplicon context sequences. **Table S3.** Rhesus-specific TaqMan assays to assess DNA methylation in bisulfite-converted DNA. **Fig. S1.** Co-localization of mutated codons in rhesus macaque CRC compared to tumor variants in human cancers. **Fig. S2.** Chromosomal instability is a widespread feature of rhesus CRC. **Fig. S3.** Transcriptomics data suggests extracellular matrix deposition and degradation and a widely immunosuppressed microenvironment in rhesus CRCs. **Fig. S4.** Ingenuity Pathway Analysis of rhesus CRC transcriptomics. **Fig. S5.** Cohort wide changes in DNA methylation levels. **Fig. S6.** Transcription factor binding sites in the promoter regions of MLH1, CACNA1G, CDKN2A, CRABP1, and NEUROG1. **Fig. S7.** Monte-Carlo simulations of intrinsic DNA topology upon DNA methylation. **Fig. S8.** Topological departure of TFAP2A binding motif in MLH1 promoter upon experimentally confirmed DNA methylation. **Fig. S9.** Spearman correlation of clinical and molecular parameters.

**Additional file 2: File S1.** Somatic tumor variants in rhesus CRCs (78 gene panel, Excel file).

## Data Availability

DNA and RNAseq files were deposited in the Sequence Read Archive (SRA, RRID:SCR_001370) under BioProject PRJNA934967 and will be made available after a 1 year embargo from the day of publication. A curated table of somatic tumor variants in our 78 gene hotspot panel (Additional file 1: Table S1) can furthermore be found in the Supplementary Materials (Additional file 2: File S1). Further information on bioinformatics coding or applied cut-offs is available upon request. Any additional data is available from the corresponding author upon completion of a DTA.
