# Supplementary material for: Epigenetic MLH1 silencing concurs with mismatch repair deficiency in sporadic, naturally occurring colorectal cancer in rhesus macaques
Source: J Transl Med. 2024 Mar 19;22:292. doi: 10.1186/s12967-024-04869-6 (PMC10953092; doi:10.1186/s12967-024-04869-6)

Additional Material

**Epigenetic MLH1 silencing concurs with mismatch repair deficiency in sporadic, naturally occurring colorectal cancer in rhesus macaques**

Deycmar, et al., 2024

## Table S1 78 gene hotspot panel applied for tumor variant calling in rhesus CRCs

## Table S2 RT-qPCR – probe and amplicon context sequences

## Table S3 Rhesus-specific TaqMan assays to assess DNA methylation in bisulfite-converted DNA

## File S1 Somatic tumor variants in rhesus CRCs (78 gene panel, Excel file)

## Fig. S1 Co-localization of mutated codons in rhesus macaque CRC compared to tumor variants in human cancers

## Fig. S2 Chromosomal instability is a widespread feature of rhesus CRC

Fig. S3 Transcriptomics data suggests extracellular matrix deposition and degradation and a widely immunosuppressed microenvironment in rhesus CRCs

Fig. S4 Ingenuity Pathway Analysis of rhesus CRC transcriptomics

Fig. S5 Cohort wide changes in DNA methylation levels

Fig. S6 Transcription factor binding sites in the promoter regions of MLH1, CACNA1G, CDKN2A, CRABP1, and NEUROG1

Fig. S7 Monte-Carlo simulations of intrinsic DNA topology upon
DNA methylation

Fig. S8 Topological departure of TFAP2A binding motif in MLH1 promoter upon
experimentally confirmed DNA methylation

Fig. S9 Spearman correlation of clinical and molecular parameters

## Table S1 78 gene hotspot panel applied for tumor variant calling in rhesus CRCs
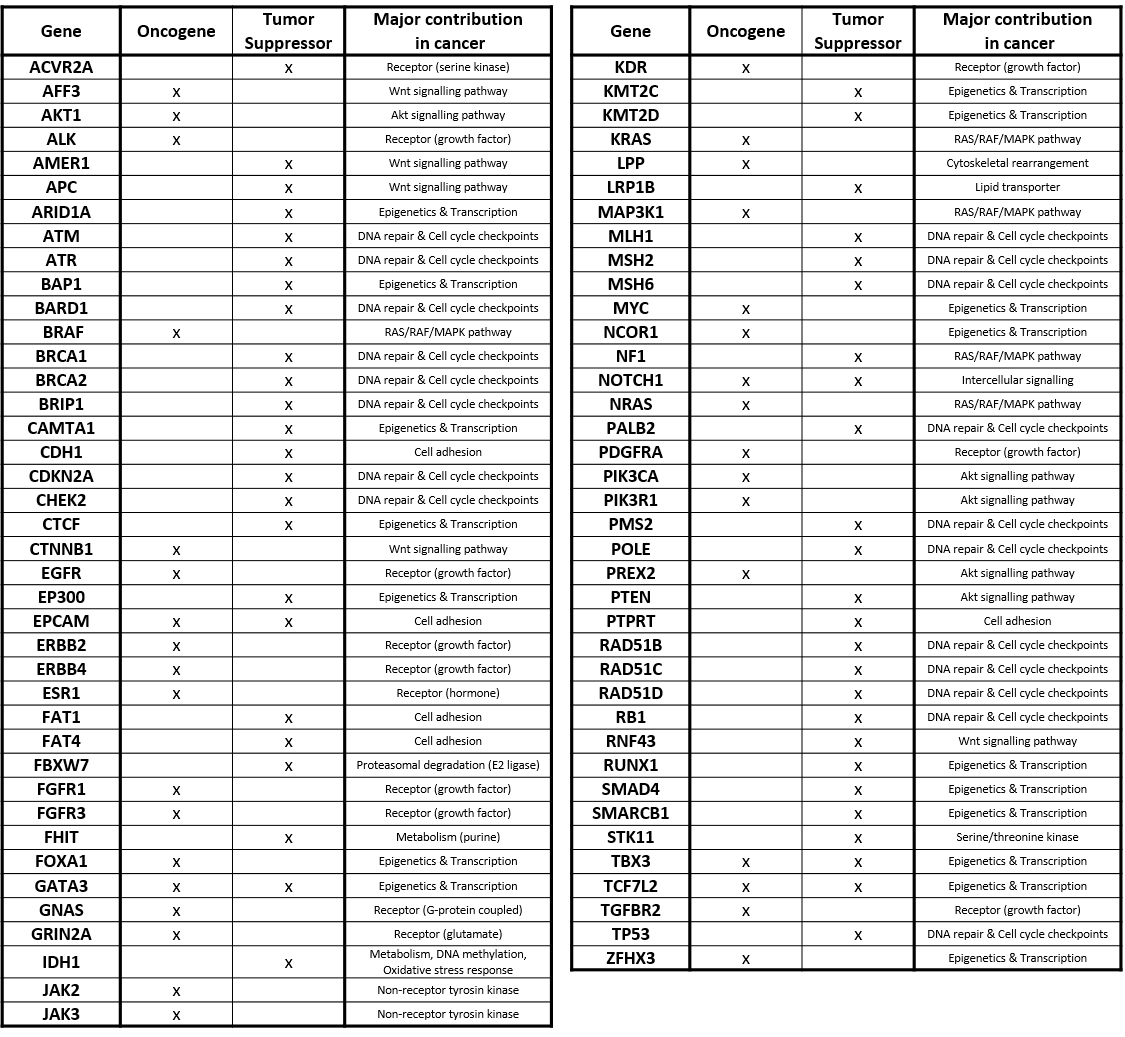


## Table S2 RT-qPCR – probe and amplicon context sequences

Context sequences, in compliance with MIQE guidelines, for our Taqman assays used to quantify mMLH1 expression. A custom assay for macaque mGAPDH was utilized as a reference to calculate ddCt values.

| MLH1 (Rh02827580_m1) |
| --- |
| **Probe context sequence** |
| >XM_015131399.2:1079-1103 PREDICTED: Macaca mulatta mutL homolog 1 (MLH1), transcript variant X1, mRNA  ACACCCATTCCTGTACCTCAGTTTA  **Amplicon context sequence** |
| >XM_015131399.2:951-1231 PREDICTED: Macaca mulatta mutL homolog 1 (MLH1), transcript variant X1, mRNA  ATATCCAATGCAAACTACTCAGTGAAGAAATGCATCTTCTTACTCTTCATCAACCATCGTCTGGTAGAATCAACTTCCTTGAGAAAAGCCATAGAAACAGTGTATGCAGCCTACTTGCCCAAAAACACACACCCATTCCTGTACCTCAGTTTAGAAATCAGTCCCCAGAATGTGGATGTTAATGTGCACCCCACAAAGCATGAAGTTCACTTCCTGCACGAGGAGAGCATCCTGGAGCGGGTGCAACAGCACATCGAGAGCAAGCTCCTGGGCTCCAATTC |
| GAPDH (custom) |
| **Probe context sequence** |
| >XM_028828779.1:1129-1152 PREDICTED: Macaca mulatta glyceraldehyde-3-phosphate dehydrogenase (GAPDH), transcript variant X1, mRNA  GCTCATTTCCTGGTATGACAACGA |
| **Amplicon context sequence** |
| >XM_028828779.1:1105-1173 PREDICTED: Macaca mulatta glyceraldehyde-3-phosphate dehydrogenase (GAPDH), transcript variant X1, mRNA  TGCCCTCAACGACCACTTTGTCAAGCTCATTTCCTGGTATGACAACGAATTTGGCTACAGCAACAGGGT |

## Table S3 Rhesus-specific TaqMan assays to assess DNA methylation in bisulfite-converted DNA

TaqMan assays were designed to probe 2-4 CpG’s in predicted CpG islands within the range of -1,500bp 5’UTR of our target genes and up to +500bp into the gene. We utilized IGV [60] to obtain the sequence and for post-design visualization and manual correction with Mmul_10 [55] as reference genome. CpG island prediction and assay design was performed with MethPrimer 2.0 [65]. In case of absent gene annotation (CDKN2A) we utilized the human gene homologue (hg38) to delineate 5’UTR and gene start, confirmed sequence homology manually, and designed the assay as mentioned above.


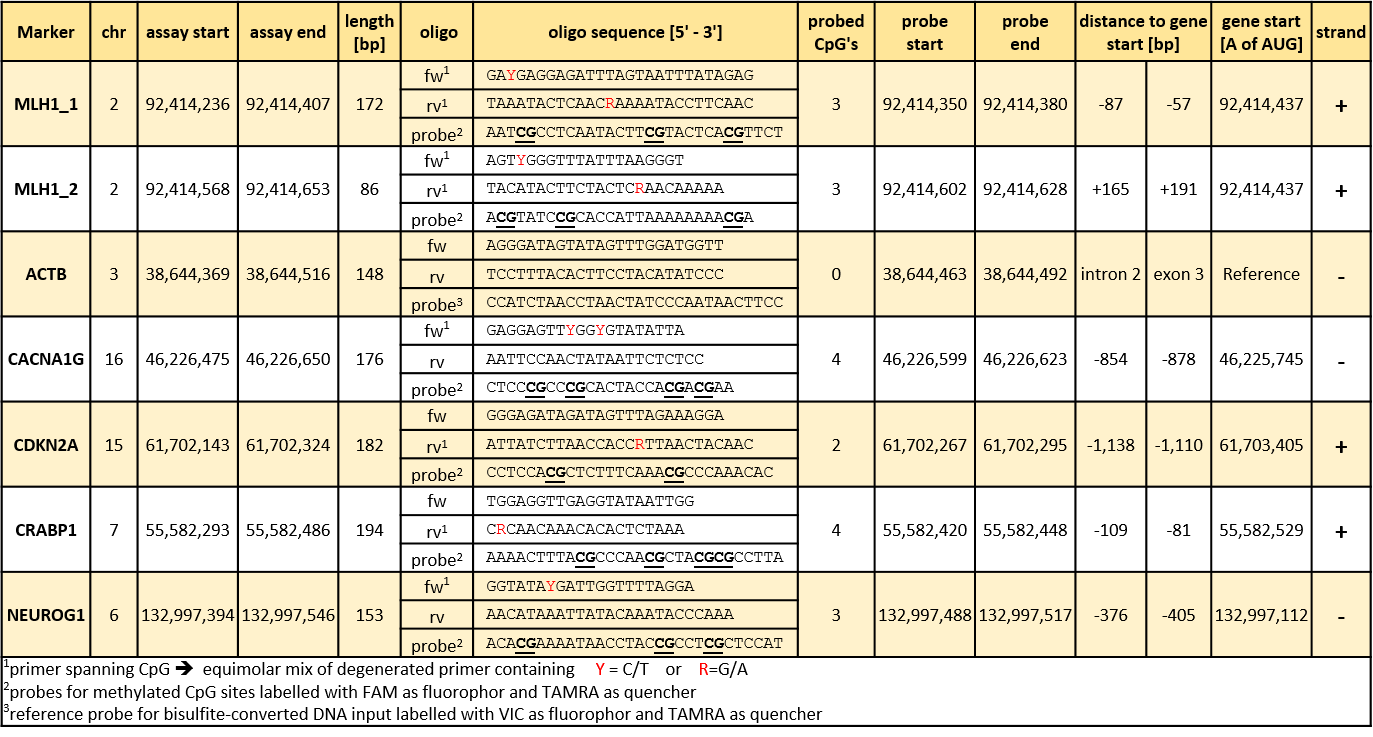


## File S1 Somatic tumor variants in rhesus CRCs (78 gene panel, Excel file)

- Attached as a tabular Excel file

## Fig. S1 Co-localization of mutated codons in rhesus macaque CRC compared to tumor variants in human cancers


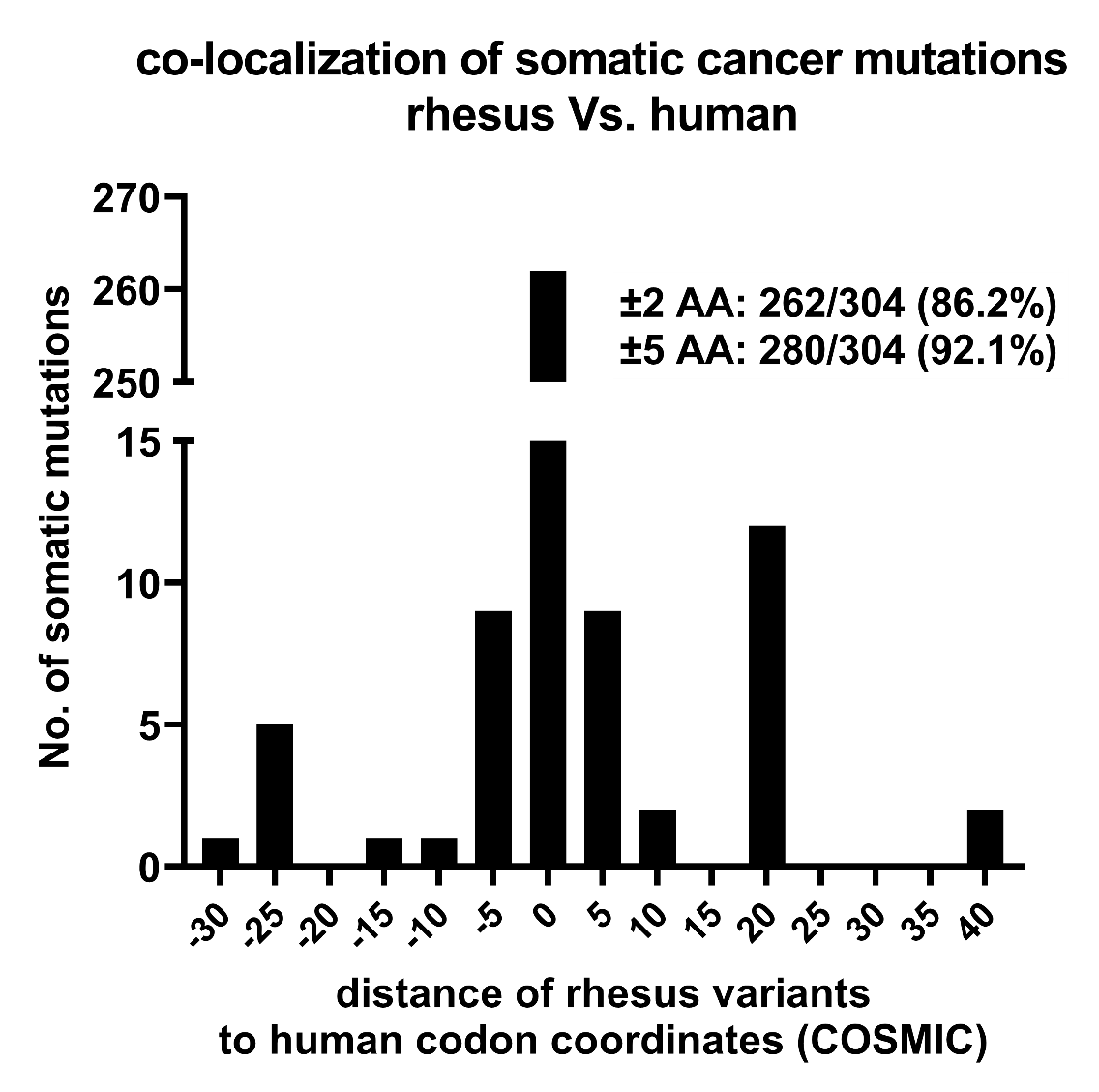


## Fig. S2 Chromosomal instability is a widespread feature of rhesus CRCs

Analysis of our whole exome data revealed aneuploidies (numerical CIN) and chromosomal alterations (structural CIN) such as loss (red arrow) or amplification (blue arrow) of entire chromosomal arms or limited sections. Moreover, we observed alternating allele frequencies (yellow arrow), suggesting loss of heterozygosity (e.g. region of chr3 and the complete chr19) in all examined CRCs.
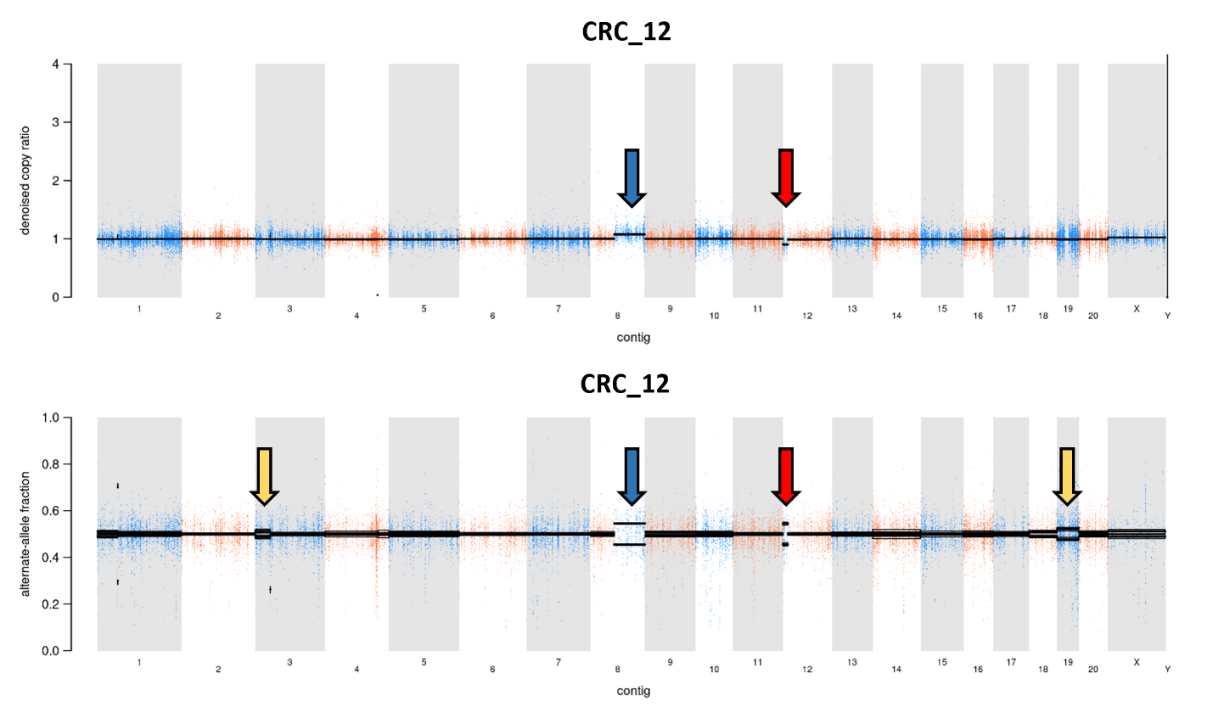

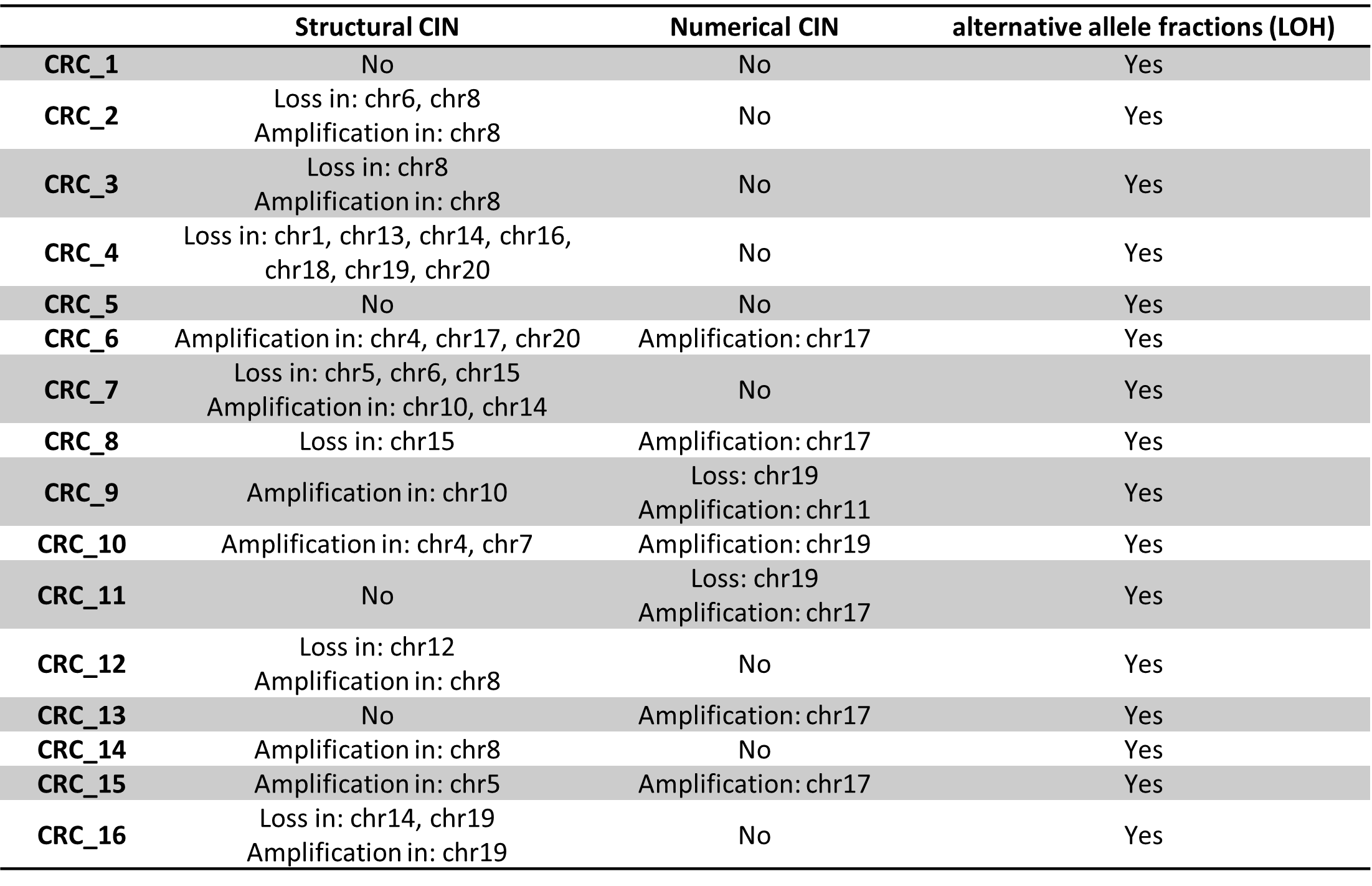


## Fig. S3 Transcriptomics data suggests extracellular matrix restructuring and a widely immunosuppressed microenvironment in rhesus CRCs

Compilation of differentially expressed genes reveals a strong overexpression of collagen genes and elastin as well as cancer-associated fibroblast (CAF) marker FAP in rhesus CRCs. Moreover, MMPs are highly overexpressed as well as TIMP1, a pro-CAF and pro-tumorigenic inhibitor of certain MMPs. This massive fibrotic response closely aligns with the histopathological evaluation. Additionally, there is a strong suppression of immune related genes such as CD226, CD244, and particularly CD8A, GZMA, and GZMB. Moreover, immunosuppressive checkpoint B7-H3 is upregulated while activation marker CD69 is downregulated. This immunodepleted pattern continues with downregulation of CD24, NKG2D, CD209, CD163L1, CD200R1, and CD200R1L. Interestingly, TLR2 and downstream targets IL1B, IL8, and IL23A are upregulated, suggesting a potential role in immunosuppression and chemoresistance in rhesus CRCs.


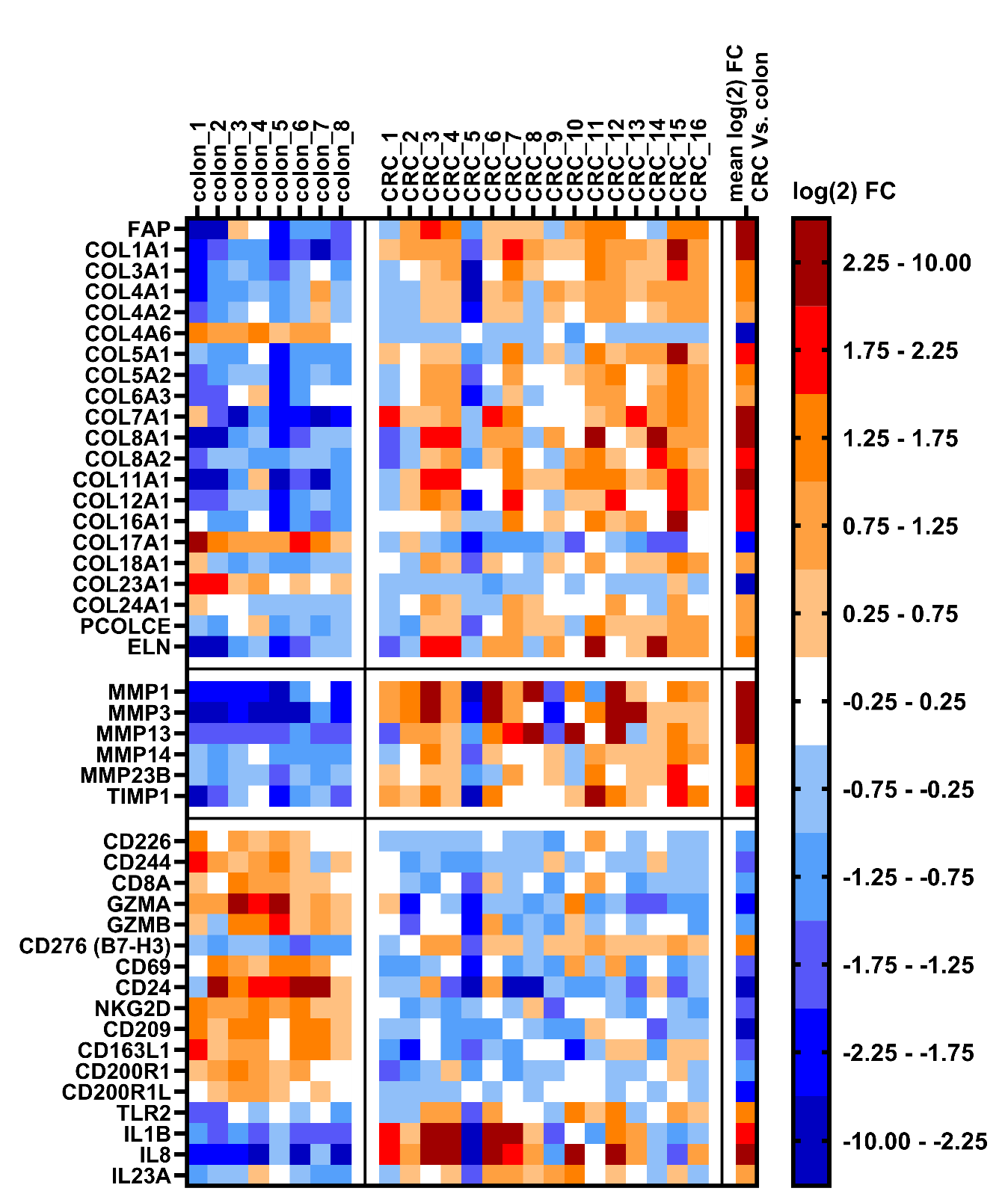


## Fig. S4 Ingenuity Pathway Analysis of rhesus CRC transcriptomics

Differentially expressed genes were inserted into Ingenuity Pathway Analysis (Qiagen, Vers. 73620684) for an unbiased comparison with annotated human transcriptomics data using right-tailed Fisher’s Exact Test. As a result, the most prominent hit was “non-melanoma solid tumor” (p=8.98*10^-63^), followed by “malignant solid tumor” (p=1.01*10^-61^), “nonhematological solid tumor” (p=1.09*10^-61^), “epithelial neoplasm” (1.39*10^-61^), and “carcinoma” (p=2.96*10^-61^). Thus, we consider the transcriptional profile of rhesus CRCs as highly comparable to human cancers.


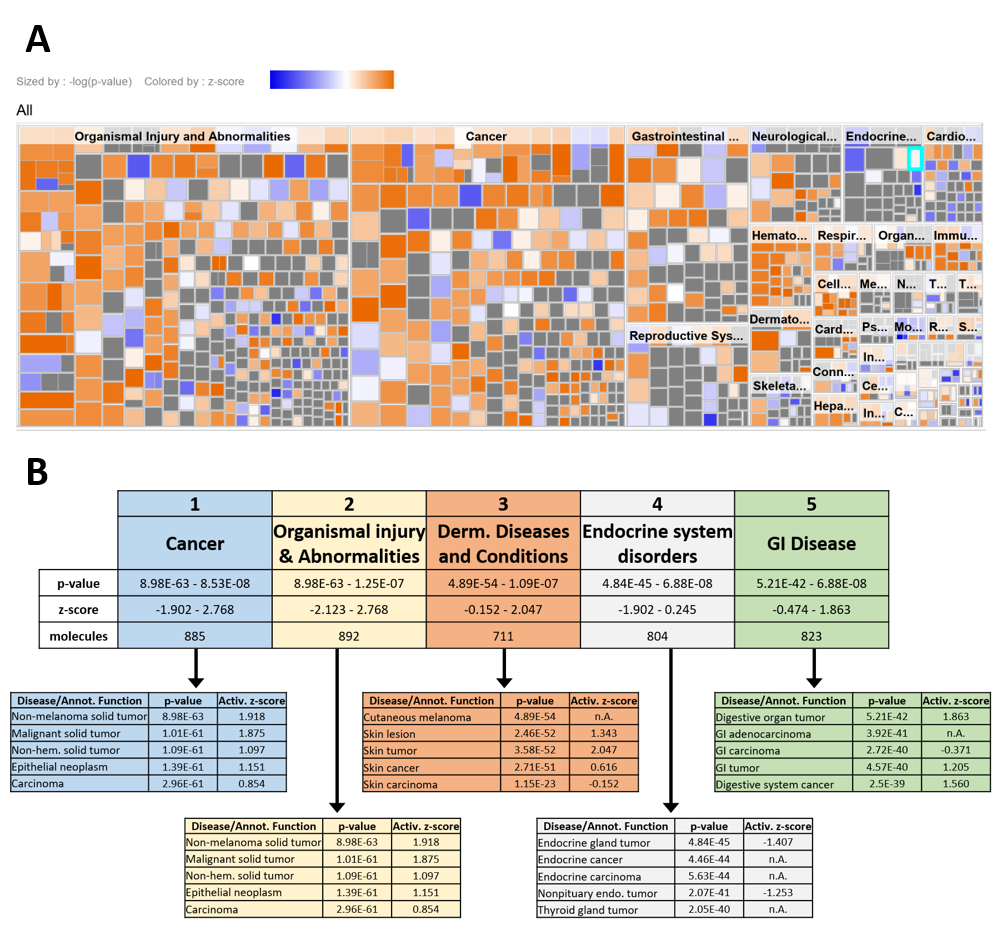


## Fig. S5 Cohort wide changes in DNA methylation levels

(A) The increase in MLH1 methylation was omnipresent in the entire cohort and (B) could be corroborated by a second, independently designed probe. (C-F) No cohort wide increase was statistically significant for the other markers CACNA1G, CDKN2A, CRABP1, or NEUROG1. Statistical analysis was performed by nonparametric Mann-Whitney U test with a p<0.05 considered as statistically significant.


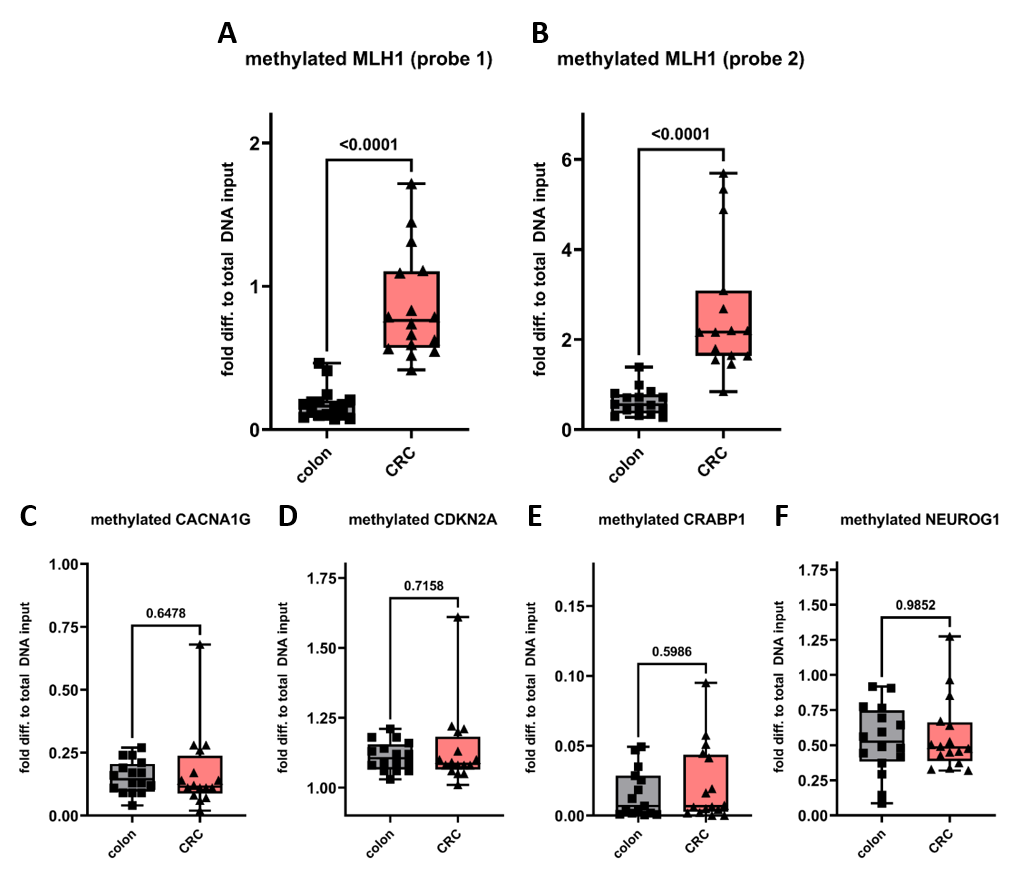


## Fig. S6 Transcription factor binding sites in the promoter regions of MLH1, CACNA1G, CDKN2A, CRABP1, and NEUROG1

We utilized ConTra v3 [42] for prediction of TF binding sites conserved in humans and rhesus macaques. We probed 1,500bp of the promoter region and plotted those TF bindings site closest to the probe-binding region assessed by our DNA methylation-specific assays. Probe-binding regions are marked in red and TF bindings sites stacked if multiple TF are predicted to bind in the same region.


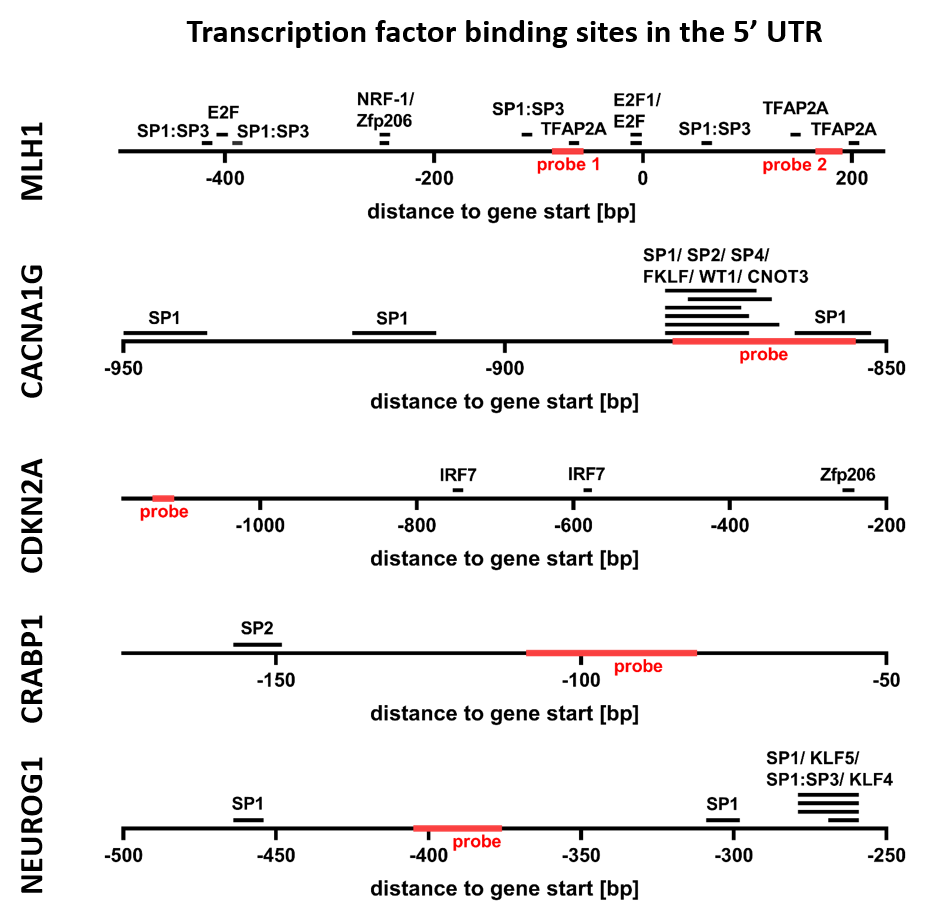


## Fig. S7 Monte-Carlo simulations of intrinsic DNA topology upon DNA methylation

We obtained DNA sequences from IGV (Mmul_10) representing the probe-binding sequence of our methylation-specific assays and the bordering 20bp in both directions. Four intrinsic DNA topology features (Minor Groove Width, Propeller Twist, Helix Twist, and Roll) were predicted with (blue line) and without (black line) cytosine methylation. Methylation sites probed by our qPCR assays are denoted with red vertical lines.


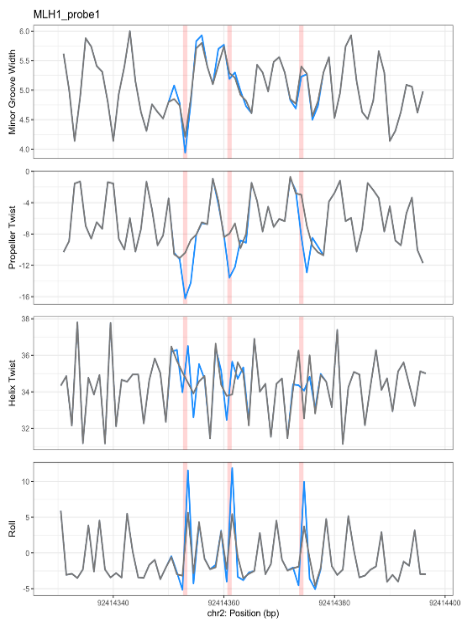

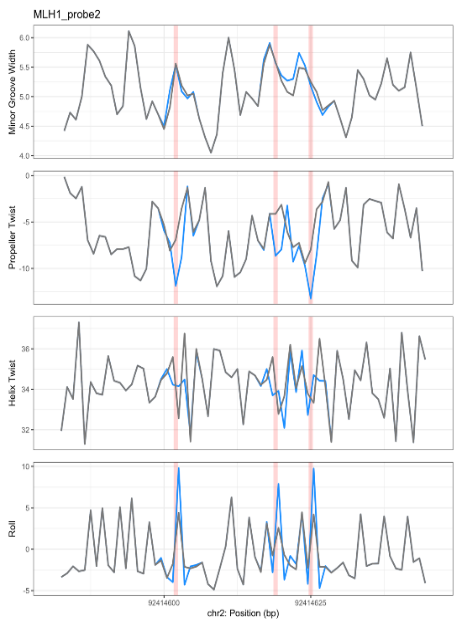

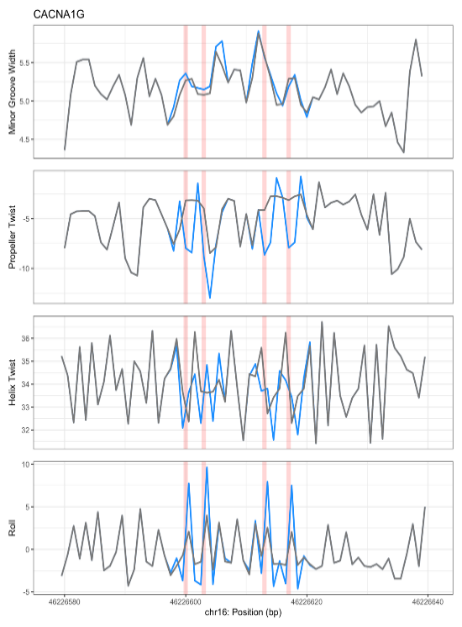

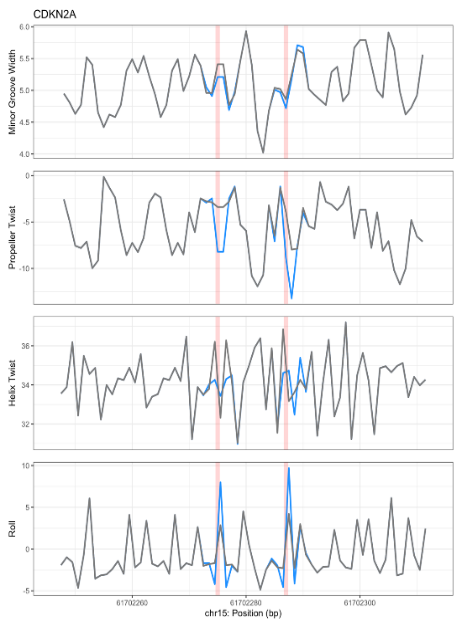

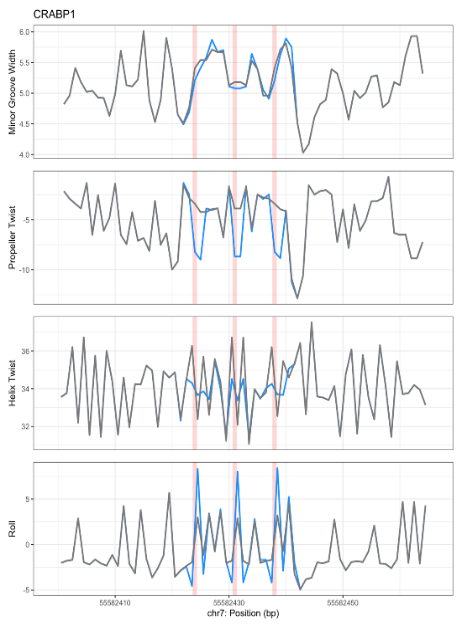

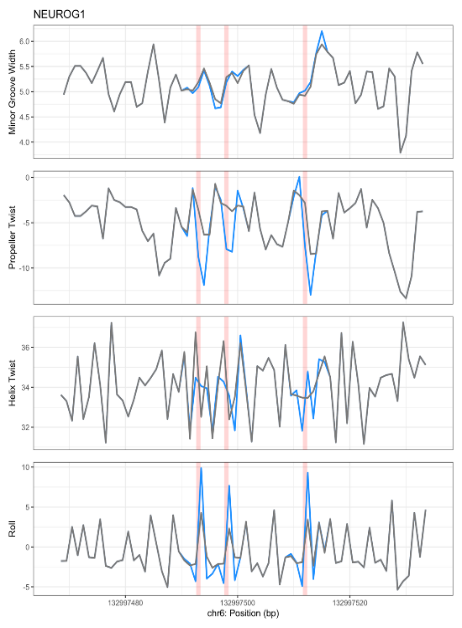


## Fig. S8 Topological departure of TFAP2A binding motif in MLH1 promoter upon experimentally confirmed DNA methylation

A TFAP2A binding site in the MLH1 promoter region was predicted to overlap with one of the qPCR probes (MLH1 probe 1). Propeller twist and Roll features for the methylated (blue line) and unmethylated (black line) probe sequence are shown. Red vertical lines denote location of CpGs and potential methylation. Known TFAP2A binding sequences- (n=15,967) were downloaded from Jaspar and the 10^th^-90^th^ and the 25^th^ to 75^th^ percentiles for each shape feature, at each position, are illustrated in gray. Methylation of the probe sequence shows departure from the topological profile of known TFAP2A binding sequences. This topological disruption illustrates a potential mechanism for TF binding interference in the presence of methylation.


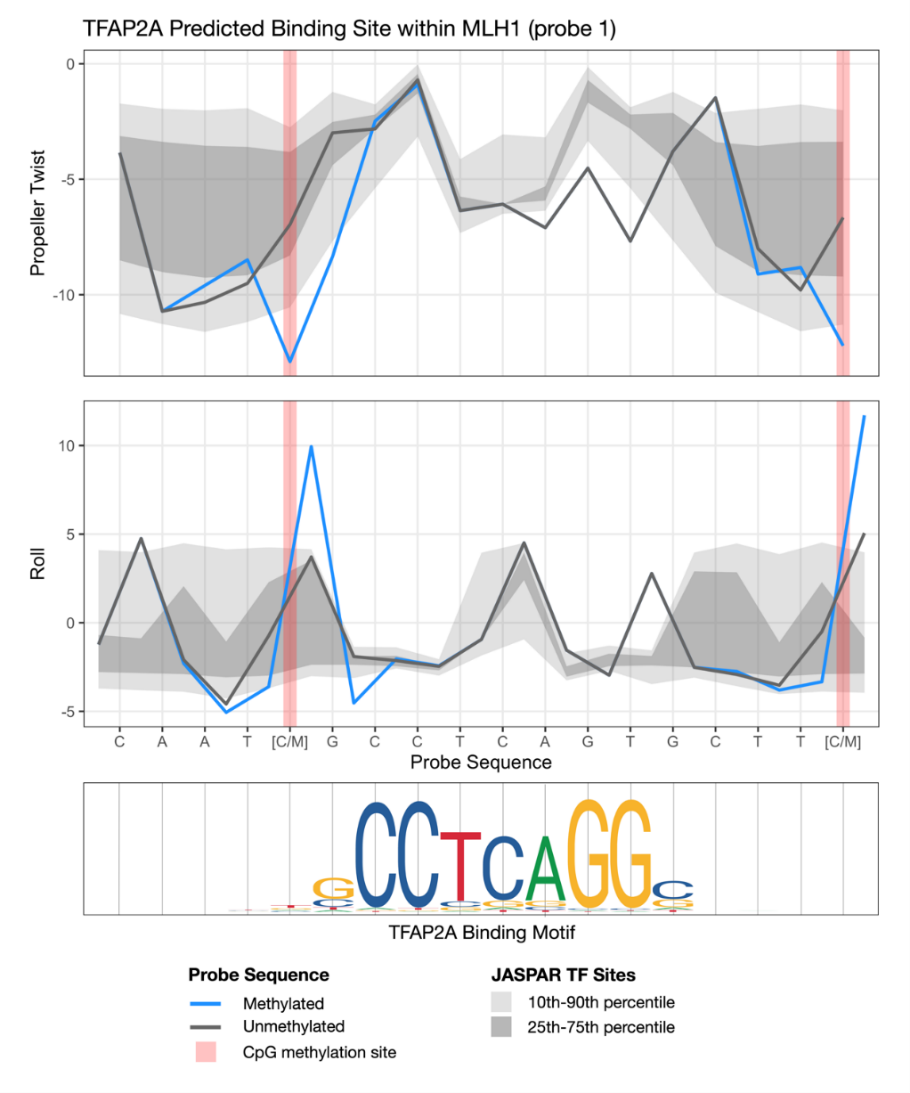


## Fig. S9 Spearman correlation of clinical and molecular parameters

We compared versatile parameters by nonparametric Spearman correlation and considered a p<0.05 as statistically significant (red squares). TP53 is a key player in the maintenance of genomic stability and correlates with the no. of instable MSI loci and TMB in rhesus CRCs. Another interesting finding is the correlation of ARID1A mutations with CIMP status, a connection similarly described for human cancers.


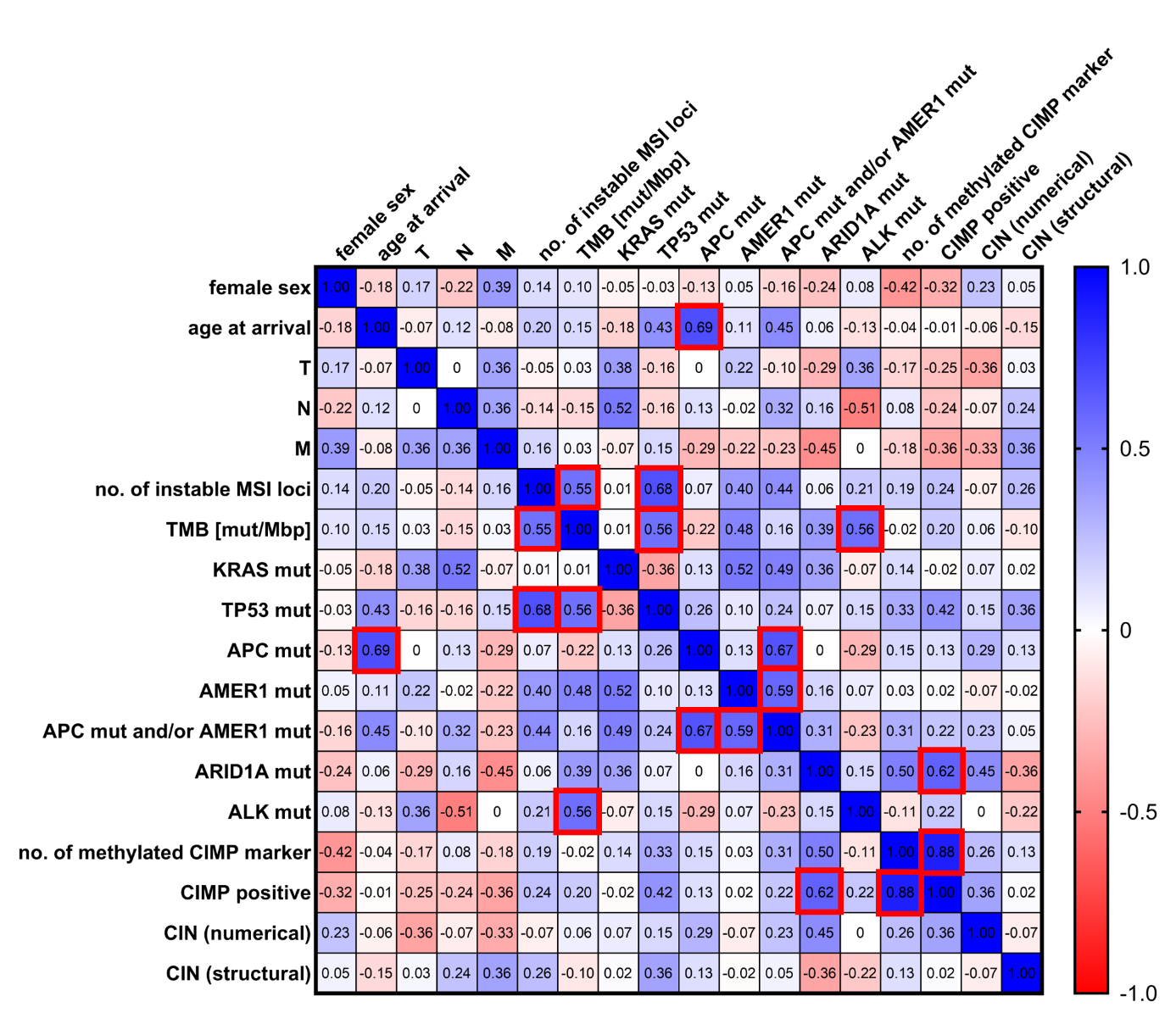

Supplement: Supplementary file 1 — Additional file 1: Table S1. 78 gene hotspot panel applied for tumor variant calling in rhesus CRCs. Table S2. RT-qPCR – probe and amplicon context sequences. Table S3. Rhesus-specific TaqMan assays to assess DNA methylation in bisulfite-converted DNA. Fig. S1. Co-localization of mutated codons in rhesus macaque CRC compared to tumor variants in human cancers. Fig. S2. Chromosomal instability is a widespread feature of rhesus CRC. Fig. S3. Transcriptomics data suggests extracellular matrix deposition and degradation and a widely immunosuppressed microenvironment in rhesus CRCs. Fig. S4. Ingenuity Pathway Analysis of rhesus CRC transcriptomics. Fig. S5. Cohort wide changes in DNA methylation levels. Fig. S6. Transcription factor binding sites in the promoter regions of MLH1, CACNA1G, CDKN2A, CRABP1, and NEUROG1. Fig. S7. Monte-Carlo simulations of intrinsic DNA topology upon DNA methylation. Fig. S8. Topological departure of TFAP2A binding motif in MLH1 promoter upon experimentally confirmed DNA methylation. Fig. S9. Spearman correlation of clinical and molecular parameters. [file 12967_2024_4869_MOESM1_ESM.docx]
